# YOLOv8's advancements in tuberculosis identification from chest images

**DOI:** 10.3389/fdata.2024.1401981

**Published:** 2024-06-27

**Authors:** Mohamudha Parveen Rahamathulla, W. R. Sam Emmanuel, A. Bindhu, Mohamed Mustaq Ahmed

**Affiliations:** ^1^Department of Basic Medical Science, College of Medicine, Prince Sattam bin Abdulaziz University, Al-Kharj, Saudi Arabia; ^2^Department of Computer Science and Research Centre, Nesamony Memorial Christian College, Marthandam, Tamil Nadu, India; ^3^Department of Computer Science, Infant Jesus College of Arts and Science for Women, Mulagumoodu, Tamil Nadu, India; ^4^Department of Information Technology, The New College, Chennai, Tamil Nadu, India

**Keywords:** tuberculosis, Yolov8 model, Selection Focal Fusion block, attention mechanism and object detection, computer-aided diagnosis

## Abstract

Tuberculosis (TB) is a chronic and pathogenic disease that leads to life-threatening situations like death. Many people have been affected by TB owing to inaccuracy, late diagnosis, and deficiency of treatment. The early detection of TB is important to protect people from the severity of the disease and its threatening consequences. Traditionally, different manual methods have been used for TB prediction, such as chest X-rays and CT scans. Nevertheless, these approaches are identified as time-consuming and ineffective for achieving optimal results. To resolve this problem, several researchers have focused on TB prediction. Conversely, it results in a lack of accuracy, overfitting of data, and speed. For improving TB prediction, the proposed research employs the Selection Focal Fusion (SFF) block in the You Look Only Once v8 (YOLOv8, Ultralytics software company, Los Angeles, United States) object detection model with attention mechanism through the Kaggle TBX-11k dataset. The YOLOv8 is used for its ability to detect multiple objects in a single pass. However, it struggles with small objects and finds it impossible to perform fine-grained classifications. To evade this problem, the proposed research incorporates the SFF technique to improve detection performance and decrease small object missed detection rates. Correspondingly, the efficacy of the projected mechanism is calculated utilizing various performance metrics such as recall, precision, F1Score, and mean Average Precision (mAP) to estimate the performance of the proposed framework. Furthermore, the comparison of existing models reveals the efficiency of the proposed research. The present research is envisioned to contribute to the medical world and assist radiologists in identifying tuberculosis using the YOLOv8 model to obtain an optimal outcome.

## 1 Introduction

Globally, tuberculosis (TB) is a severe public health concern caused by Mycobacterium tuberculosis. It significantly affects the human lungs (pulmonary TB), (Kotei and Thirunavukarasu, 2024) but also other parts of the body such as the spine, brain, and kidneys (extrapulmonary TB) (Wang et al., 2024). Usually, TB spreads by air; it transmits from one person to another through coughs and sneezes. It can be detected through different tests, like blood tests, sputum tests, imaging studies, or skin tests (Maipan-uku et al., 2024). It is crucial to identify it at an early stage and control it with proper treatment. Besides, bacteria are considered the most common cause of TB in healthcare settings (Amin et al., 2024). Henceforth, TB is considered to be the leading cause of death among infectious diseases. Chest radiography is playing a vital role in detecting TB at a low cost. Resource-constrained countries are bearing the burden of TB. However, the insufficient availability of expert readers is considered a major concern hampering the application of chest radiography (Hwang et al., 2024). To evade the prediction problem, an effective disease prediction mechanism is needed to avoid the consequences. Recently, most researchers have utilized Artificial Intelligence (AI)-related (Acharya et al., 2024) technology for the disease prediction of TB and non-TB for the capability of better cost-saving techniques and greater scalability. Furthermore, AI technology can systematize prediction problems and monitor the data efficiently. It can identify abnormalities in the data while in access. These AI benefits with Machine Learning (ML) and Deep Learning (DL) methods (Rahman et al., 2020) provide several benefits for classification mechanisms in TB prediction at various stages.

Congruently, several prevailing models have attempted to accomplish better TB and non-TB prediction. For instance, the existing model, a fine-tuning deep neural network, has been used to perform lesion detection for tuberculosis. The utilized task dataset and the results have shown that the convolutional neural network (CNN) model has attained a better mean Area Under Curve (AUC), which represents the better efficiency of the prevailing model (Lu et al., 2022). Similarly, the conventional model has deployed detection mechanisms for Pulmonary Tuberculosis lesions and utilized the TBX-11 dataset. The outcomes of the conventional model have attained 0.5 Intersection Over Union (IoU) of average precision, and the recall rate has attained 77.6% (An et al., 2022). Correspondingly, Computer-Aided Diagnosis (CAD) utilizing computer vision methods and advanced DL models has been used to improve the accuracy using the YOLOv7 object detection architecture. It has used the TBX-11 dataset and employed data augmentation and class weighting techniques to notice the imbalance present in the dataset. The promising results have shown that the conventional model has attained 0.587 mean Average Precision (mAP) (Bista et al., 2023). Likewise, the prevailing model has deployed a TB detection framework and utilized the Montgomery and Shenzhen datasets. The conventional model was evaluated with a *k*-fold cross-validation technique and attained 0.97 and 0.99 of AUC for the Montgomery and Shenzhen datasets, respectively (Ayaz et al., 2021). Similarly, the prevailing model has developed DL classification and segmentation models (Iqbal et al., 2022) for precise and accurate detection of TB on chest X-ray (CXR) images with the vision of infection utilizing Gradient-weighted Class Activation Mapping (Grad-CAM) heat maps. It has utilized the NIAID TB portal dataset (Ekins and Freundlich, 2011; Acharya et al., 2022) and has applied the Xception model. The results have attained better recall, F1 score, accuracy, and precision (Sharma et al., 2023). Accordingly, classical models have accomplished satisfactory results, but they lack a few limitations, such as mAP, recall, precision, speed, and overfitting of data.

To solve this problem, the proposed research utilizes certain procedures to improve the performance of the YOLOv8 model for TB and non-TB prediction. Initially, the TBX-11 dataset is loaded into the mechanism where the images are utilized to enhance the proposed model's performance. It is processed with image label pre-processing to improve the consistency and efficiency of the image data. Correspondingly, after the image processing, it is forwarded to pre-processing with augmentation. It transfers the utilized data to augmented samples to improve efficiency. Then, processed data is divided into training, validating, and testing data to train the present model and evaluate its performance. Accordingly, the training data are used for the prediction with Selection Focal Fusion (SFF) in the YOLOv8 model with an attention mechanism. After the prediction mechanism, the validating set is used for performance calculation in the present framework. Additionally, the efficiency of the projected model is calculated utilizing performance metrics to examine the proposed prediction performance. The major contribution of the projected model is discussed in the following:

- To utilize YOLOv8 for the prediction of tuberculosis with the Kaggle TBX-11k dataset to enhance the accuracy and computation in the present research.
- To employ the SFF technique in the YOLOv8 model with attention mechanism for prediction of active TB, latent TB, healthy and sick, but non-TB for enhancing the prediction efficiency.
- To calculate the efficiency of the projected system with performance metrics such as precision, recall, mAP, and F1 score.

### 1.1 Paper organization

The paper's organization is significant for communicating the research findings in a coherent manner. It is organized on the basis of analyzing the existing methods and the approaches applied under the prediction of Active Tuberculosis (ATB), latent TB, and non-TB in Section 2. Section 3 provides the proposed methodology for the current research. Moreover, the results accomplished through the current system are illustrated in Section 4. The outcomes of future research using the current approach are depicted in Section 5.

## 2 Literature review

This section explains the analysis of various existing models of ML and DL techniques for the prediction of TB and non-attack in the non-TB classification systems. Furthermore, the problem mentioned in the prevailing research is also identified. The conventional model has evaluated its diagnostic performance utilizing ML techniques (Anand et al., 2021) in differentiating ATB from Latent Tuberculosis Infection (LTBI). It has employed the Gradient Boostng Machine (GBM) model for accurate identification of ATB. The results have shown that the GBM model has attained 89.81% accuracy (Luo et al., 2021). Similarly, the prevailing model has detected Pulmonary Tuberculosis (PTB) and Extrapulmonary Tuberculosis (EPTB) using ML algorithms. It has employed clinical and imaging data from hospitals and incorporated the Decision Tree algorithm. The result has shown that the Decision Tree (DT) algorithm has attained 95.5% classification accuracy (Kaur and Sharma, 2021). In the same way, the existing model has demonstrated a TB detection mechanism (Heyckendorf et al., 2021) utilizing advanced DL models. It has utilized EfficientNetB3 and the CNN model (Dey et al., 2022) for the classification, along with the available CXR dataset. The result has shown that the model has attained 98.7% accuracy (Nafisah and Muhammad, 2024). Correspondingly, the conventional model has diagnosed the ATB from multiplex serological data. It has utilized Machine Intelligence Learning Optimiser (MILO) for the prediction performance. It has incorporated secondary and tertiary datasets and has resulted in 86% accuracy (Rashidi et al., 2021). Contrarily, the existing model has enhanced the weight voting ensemble learning method to aid in diagnostic development for predicting TB infection at an early stage. It has used TB gene expression data for diagnosis. The results have shown that ensemble classifiers, Support Vector machine (SVM), and Naive Bayes (NB) have attained 95, 92, and 87% accuracy, respectively (Osamor and Okezie, 2021).

Congruently, the conventional system has presented a solution for identifying TB by utilizing Bayesian-based CNN (B-CNN). It has been evaluated with two TB benchmark datasets called Shenzhen and Montgomery. The results have revealed that B-CNN (Yusoff et al., 2021) has attained 86.46 and 96.4% accuracy on both datasets, respectively (Abideen et al., 2020). Contrastingly, the classical model has combined clinical indicators and metabolomics along with ML for a precise identification of Smear-Negative Pulmonary Tuberculosis (SNPT) (Xie et al., 2020) and Smear-Positive Pulmonary Tuberculosis (SPPT). The outcome has shown that the model has achieved 83–93% accuracy (Hu et al., 2022). In parallel, the prevailing model has presented an automatic cough classification for TB (Sathitratanacheewin et al., 2020) and utilized ML methods such as KNN, MLP, LR, CNN, and SVM. It has used nested cross-validation (Singh et al., 2021). Among these ML methods, LR has outperformed with 84.54% accuracy (Liu et al., 2017; Pahar et al., 2021). Simultaneously, the conventional method has suggested a DL binary classifier for TB and non-TB diagnosis utilizing chest X-rays. It has employed a 2-step binary DT and has been trained through CNN on the PyTorch frame. The prevailing model has used the Shenzhen dataset, and results have shown 98 and 80% accuracy at both the first and second steps, respectively (Yoo et al., 2020). Similarly, the conventional mechanism has developed a TB and non-TB detection and Drug-Resistant Categorization Diagnosis Decision Support System (TB-DRC-DSS) (Zhu et al., 2023) using the DL ensemble model (Rajaraman and Antani, 2020) with different CNN architectures such as Dense-Net121, mobileNetV2, and EfficientNetB7. It has incorporated the Shenzhen, (Rajaraman et al., 2021) Kaggle, Montgomery, (Le et al., 2022) and Portal datasets. The results have revealed that TB-DRC-DSS has attained 92.8% accuracy (Prasitpuriprecha et al., 2022).

In contrast, the prevailing mechanism has applied automatic AI detection for screening a huge populace and has evaluated its feasibility. It has helped in diagnosing TB utilizing CXR radiographs. The outcomes of the conventional model have shown that the AI detection model has attained 85% accuracy (Nijiati et al., 2021). Similarly, the existing model has presented a deep CNN approach for diagnosing TB utilizing CXR images. It has employed a histogram matching method with CXR images (Verma et al., 2020) for improving detection and accuracy performance for TB detection. The results have shown that the respective mechanism has attained better accuracy and F1 scores (Ignatius et al., 2023). Similarly, the existing system has presented automatic TB detection using an enhanced DL model with CXR images. For the process, the respective model has used the Shenzhen China (SC) (Yang et al., 2022) and Montgomery County (MC) datasets. The results have shown that the conventional model has attained better accuracy (Simi Margarat et al., 2022). Contrarily, the prevailing model has developed CAD for the classification of TB disease using an X-ray dataset. It has incorporated an Artificical Neural Network (ANN) with SVM for better classification and has attained 94.65 accuracy (Pathak et al., 2022). Concomitantly, the prevailing model has detected TB using LightTBNet, a lightweight, efficient, and fast deep CNN (Sahlol et al., 2020) from the CXR images. It has been evaluated with two publicly available datasets. The results have shown 90.6% accuracy, a 90.7% F1 score, and a 96.1% ROC curve (Capellán-Martín et al., 2023).

Similarly, the conventional model has predicted a bacteriologic confirmation of Mycobacterium TB in children and infants. It has employed ML models for the development of prediction performance. It has used a new dataset, and the result has shown that the prevailing mechanism has attained better values in accuracy metrics (Smith et al., 2023). Contrarily, the suggested model has diagnosed TB from real-world cough recordings and has incorporated the conventional ML models for better prediction. It has utilized a huge dataset of TB and non-TB audio recordings (cough). The outcome has shown satisfactory results (Kafentzis et al., 2023). Contrastingly, the traditional model has assessed an enhancement of image in TB detection utilizing DL methods. It has evaluated image enhancement algorithms, namely, High-Frequency Emphasis Filtering (HEF), Unsharp Masking (UM), and Contrast Limited Adaptive Histogram Equalization (CLAHE). It has used a TB image dataset, and the results have revealed that the prevailing method has attained 89.92% accuracy (Munadi et al., 2020).

### 2.1 Problem identification

- Several existing studies have been focused on predicting TB and non-TB. However, limited research has focused on the ATB, healthy, LTBI, sick, and non-TB classifications (Kaur and Sharma, 2021).
- Accuracy is an important performance metric utilized to examine the model's performance. Nevertheless, existing models lack accuracy rates and prediction rates (Osamor and Okezie, 2021).
- Due to its primary cause, in TB prediction research, feature selection has been lacking in conventional research (Capellán-Martín et al., 2023).

## 3 Materials and methods

In the present world, the severity of TB disease is increasing in most countries. It affects the lives of massive numbers of people. To evade the severity of the disease and the consequences of TB, it is important to recognize the disease. Though the prevailing research is a time-consuming and expensive process, several studies are focused on TB prediction but lack detection rates, overfitting data, and accuracy. To improve the TB prediction, the respective model employed SFF in the YOLOv8 model to classify ATB, LTBI, and non-TB, sick and healthy, through the TBX-11k dataset. Congruently, it is necessary to recognize the major cause of disease for enhanced classification, as TB is the most hazardous lung disease that affects the respiratory system.

**Figure 1:**
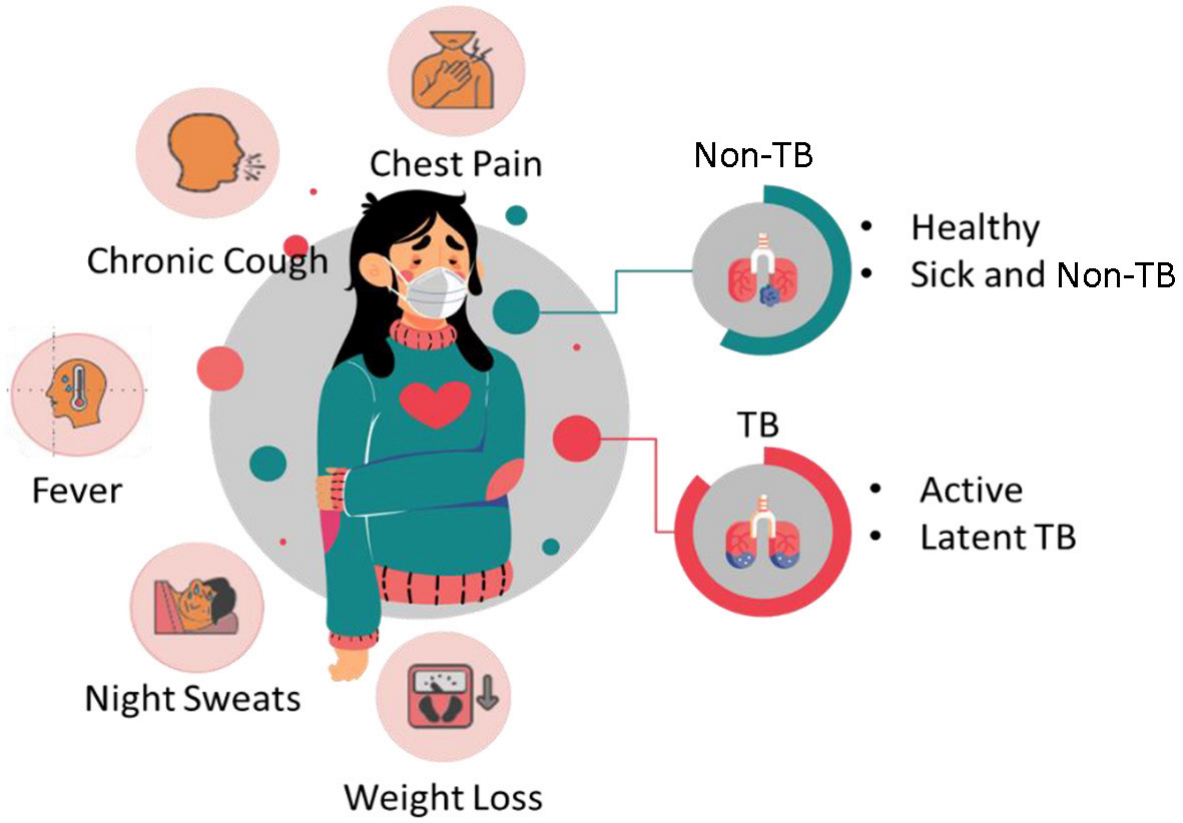
Symptoms of TB and non-TB.

Tuberculosis is a contagious bacterial infection that can be caused by bacteria called *Mycobacterium tuberculosis* complex, which is considered one of the oldest diseases. It primarily affects the lungs of humans and is the leading cause of death worldwide. Generally, TB is spread through the air while the infected person sneezes or coughs. The bacteria can be transmitted from one person to another. Some of the common symptoms of TB encompass chest pain, fatigue, chronic cough, fever, night sweats, weight loss, and coughs with blood. Figure 1 depicts the symptoms of TB and non-TB.

**Figure 2:**
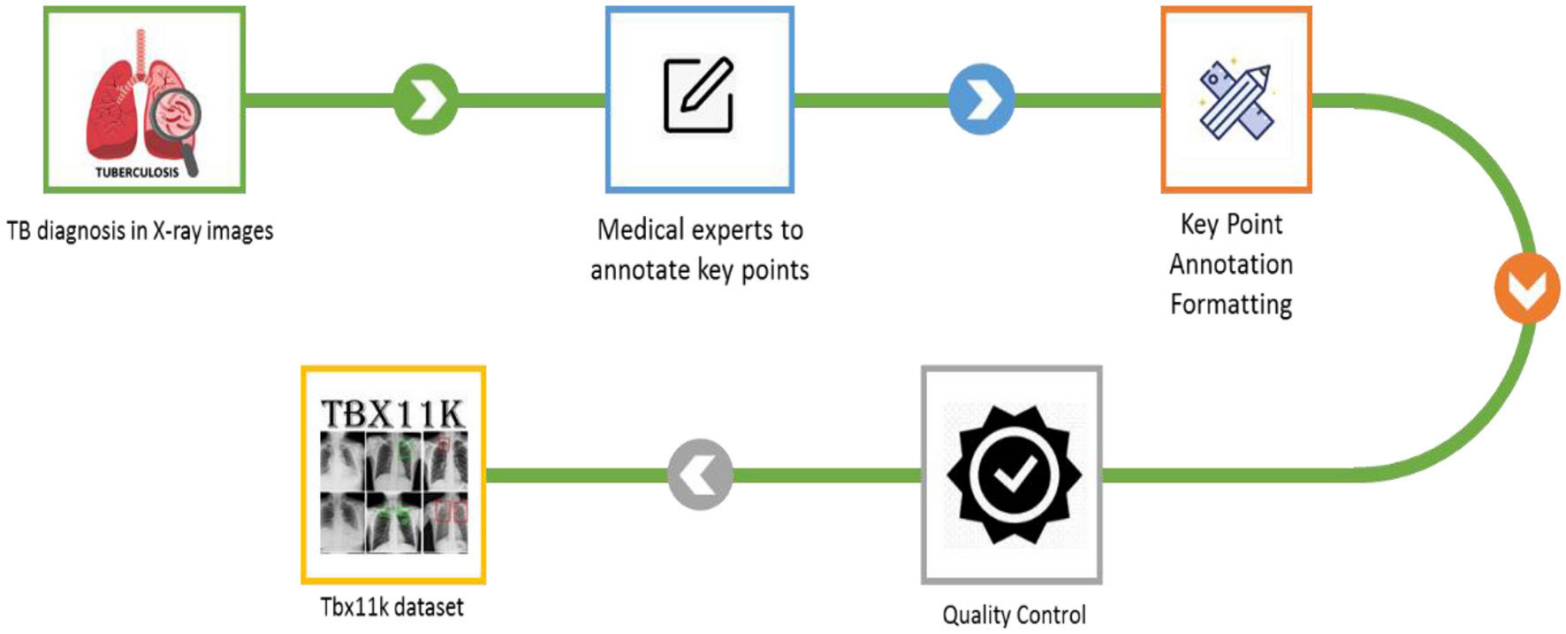
Illustrative representation of dataset creation.

Primarily, TB causes severe health problems if it is not treated or if you with a weak immune system. It leads to injury to the lungs and spreads to other parts of the organs like the spine, kidneys, and brain in some cases. The major reason for TB is *Mycobacterium tuberculosis*. It is defined as when a person inhales this bacteria, it can be transmitted to the lungs and multiply, which leads to infection. Moreover, the immune system responds by forming granulomas, which are known as small nodules that contain infection. In several cases, bacteria remain dormant for years without any symptoms. Conversely, the immune system gets weak, and infection causes illness and becomes active. Though it has severity, TB is a preventable and treatable disease. Early prediction, appropriate treatment, and medication adherence are significant in preventing and managing TB transmission. To overcome the existing research problems, the proposed model employed SFF in the YOLOv8 model to classify ATB, LTBI, and non-TB, sick and healthy, through the TBX-11k dataset. Figure 2 depicts the illustrative representation of the dataset creation.

**Figure 3:**
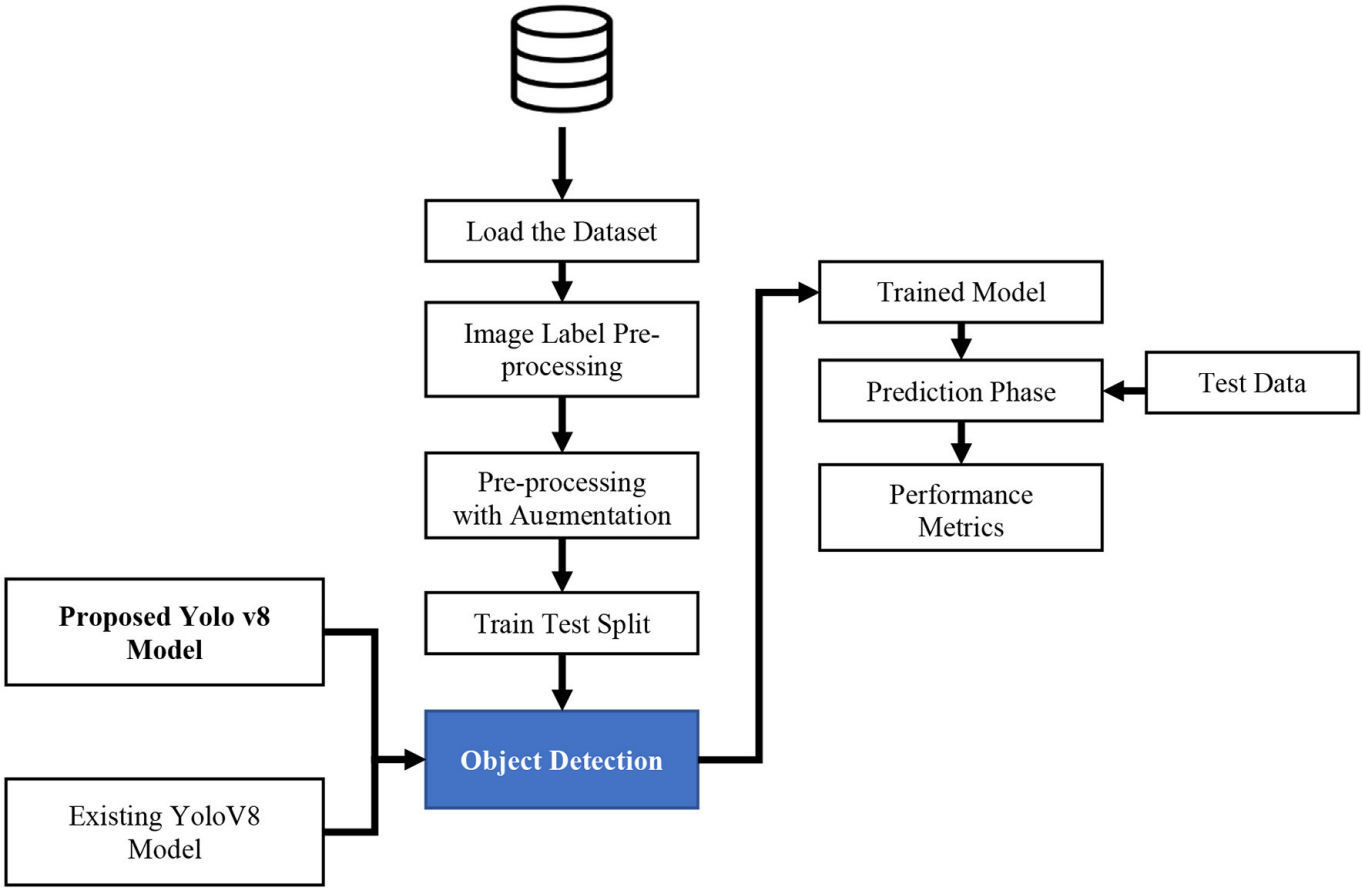
Proposed mechanism of the YOLOv8 model.

The proposed mechanism utilizes the TBX-11k dataset to predict TB through the X-ray images. It uses the advantages and incorporates SFF in YOLOv8 to enhance the prediction performance of TB. To calculate the efficiency of the proposed mechanism, performance metrics like precision, F1-score, recall, and accuracy are utilized in projected research. The proposed TB and non-TB prediction mechanisms are depicted in Figure 3.

Figure 3 signifies the present model, which is comprised of several stages, including the selection of datasets, the image label-pre-processing method, training and testing split, and prediction with SFF in the YOLOv8 model. The detailed description of each stage in the proposed system is discussed below:

### 3.1 Dataset selection

The proposed system uses the TBX-11k dataset from the Kaggle website for the prediction of TB and non-TB in X-ray images, which is publicly available. For assessing the proposed mechanism, the TBX-11k dataset is used in this model, as outlined in Figure 3. Furthermore, the dataset includes the data of both TB and non-TB, and it comprises 11,200 X-ray image samples. There are five divisions in this dataset: Sick but non-TB, Healthy, Active TB, Latent TB, and uncertain TB. It can be split into a training set, a validation set, and a testing set. The website line of the Kaggle TBX-11k dataset is given below:

https://www.kaggle.com/datasets/usmanshams/tbx-11/data.

### 3.2 Image label pre-processing

The image label pre-processing process refers to the preparation of image data for classification or labeling tasks. Typically, these steps involved normalizing and transforming the images to confirm that they were suitable for further processing. Image label pre-processing techniques include normalization, resizing, cropping, color-space conversion, and augmentation. Moreover, the pre-processing techniques support improving the consistency and efficiency of the image data and make it more suitable for the labeling and classification processes. In the projected research, the image label pre-processing method is used to convert the features of the dataset to a common scale, enhancing the accuracy and performance of the prediction.

### 3.3 Pre-processing with augmentation

The process of pre-processing with augmentation is denoted as a set of techniques that were utilized to modify and enhance the data before it was used for the training process. Augmentation methods are implemented to improve the quantity and diversity of training data that enhance the generalization ability and performance of the model. Moreover, the process involved relating different modifications and transformations to existing data and creating augmented samples. Transformations such as scaling, rotating, cropping, adding noise, flipping, and changing the contrast and brightness of images.

### 3.4 Data splitting

In the YOLOv8 model, data splitting is the process of dividing the dataset into discrete subsets for processes such as training, validation, and testing. It is a common activity in ML for evaluating the model's performance to prevent overfitting and unseen data. The training set is utilized to train the YOLOv8 model, and the validation set is used to tune the hyperparameters of the model and observe its performance. The testing set is to calculate the final performance of the trained model on unseen data. Table 1 depicts the hyperparameters and their values for training.

**Table 1:** Hyperparameters training.

**Hyperparameters**	**Value**
Epochs	100
Batch size	16
Image size	640
Optimizer	Auto
Momentum	0.937

### 3.5 Object detection

The YOLOv8 model is defined as an object detection algorithm. It aims to detect and classify the objects accurately. It is referred to as an improvement model on previous versions of the YOLO model that offers better accuracy and performance. Moreover, the model evaluation is based on mAP, which is considered one of the most utilized evaluation metrics for object detection. The mAP took the average precision (AP) on classes and computed them at the pre-mentioned IoU threshold. The proposed model assesses the generalization and robustness capabilities of models by mAP scores that are calculated among the diverse test scenarios, highlighting the significance of the YOLOv8 model in TB and non-TB detection.

#### 3.5.1 Conventional YOLOv8 model

**Figure 4:**
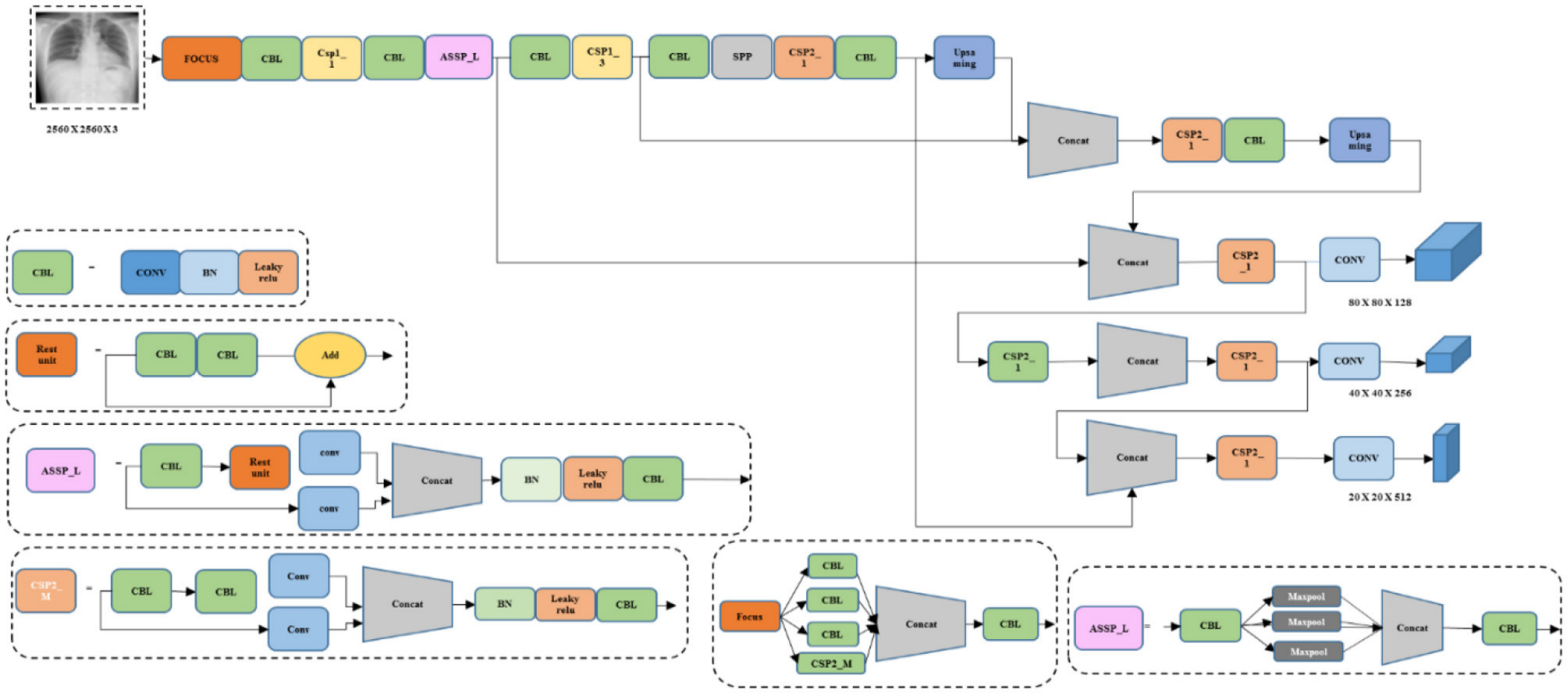
Architecture of the conventional YOLOv8 model.

YOLOv8 is a cutting-edge and advanced model that provides higher detection speed and accuracy. The YOLOv8 is known as a real-time object detection mechanism that utilizes a single Neural Network (NN) to predict class probabilities and bounding boxes directly from entire images in a single evaluation. Significantly, it is based on Darknet architecture and uses layers of convolutional layers along with skip connections to enhance speed and accuracy. The YOLOv8 model incorporates different improvements from previous versions, like advanced data augmentation techniques and feature pyramid networks, to attain better performance in the task of object detection. Figure 4 illustrates the architecture of the existing YOLOv8 model.

Figure 4 deliberates the architecture of conventional YOLOv8, a DL model. It is capable of processing the inputs in different forms, such as real-time camera images, videos, and images. Moreover, it comprises convolutional layers (conv), concatenation layers (concat), and three max pooling layers. Though it performed with satisfactory results, it lacked accuracy and speed. Thus, the proposed model incorporates several techniques to improve the performance of the proposed model.

#### 3.5.2 Proposed YOLOv8 model

**Figure 5:**
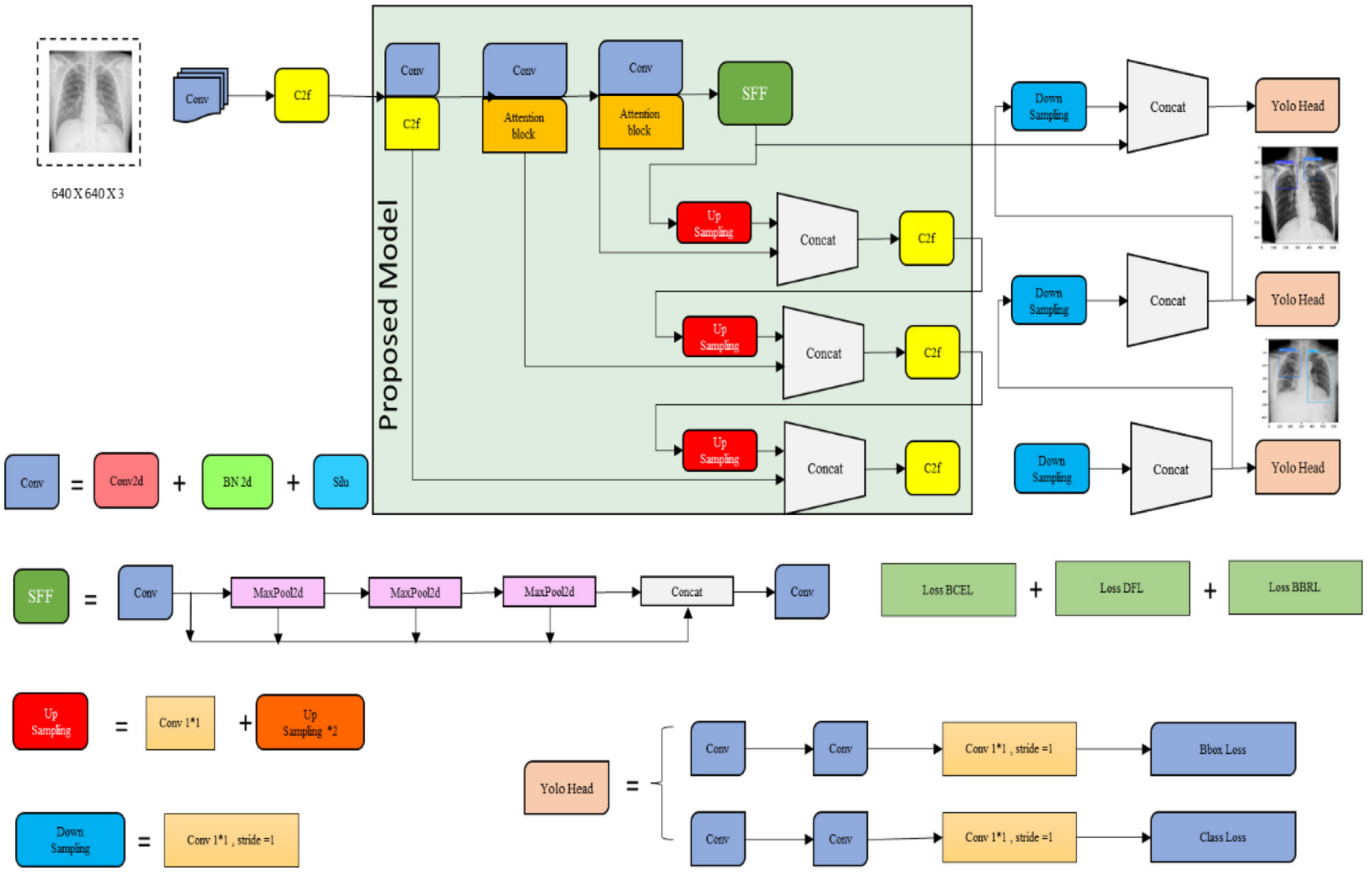
Architecture of the proposed YOLOv8 model.

The YOLOv8 model is known as a popular object detection algorithm that is utilized in system vision tasks encompassing TB detection. Primarily, it is designed for object detection; thus, the proposed research is adopted for predicting TB. This model is known for its fast inference speed, which allows for the processing of the image data rapidly and is particularly useful in the diagnosis process. The YOLOv8 model has the ability to detect multiple objects in a single pass. It is beneficial in cases where TB abnormalities or lesions are presented in various areas of the lungs or other affected organs. In addition, the model provides relatively high accuracy in object-detecting tasks, which has been trained on huge datasets, and uses Deep Neural Networks (DNN) to identify TB. Though the YOLOv8 model performs better, it struggles with small objects and finds an inability to perform fine-grained classifications. To evade this problem, the proposed research incorporates the SFF technique to improve the detection performance and decrease the small object missed detection rates. Figure 5 illustrates the architecture of the YOLOv8 model.

**Figure 6:**
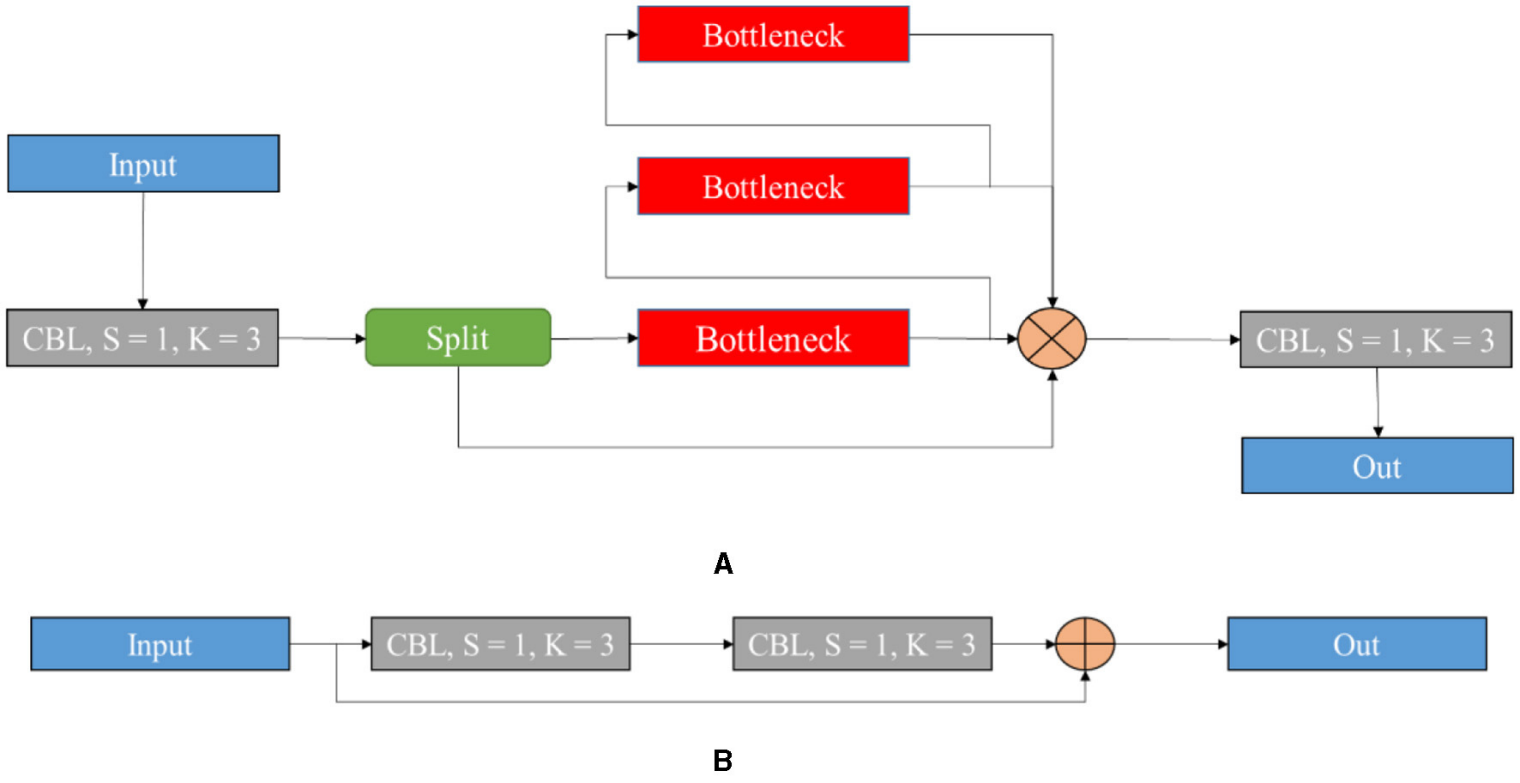
Schematic diagram of the C2f module **(A)** and bottleneck **(B)** of the present model.

Figure 5 signifies the architecture of the proposed YOLOv8 model, which incorporates improvements for high detection accuracy while maintaining high efficiency and speed. Several key modifications include the Cross-Stage Partial Bottleneck (C2f module) and the detection head, including independent branches, activation functions, and loss functions. The C2f module is incorporated to merge high-level features with contextual data effectively. It is achieved by concatenating the bottle net block output, which contains two 3 × 3 convolutions along with residual associations. The YOLOv8 model adopted the detection head for eliminating the requirement for pre-defined anchor boxes and predict the object centers directly. Figure 6 shows the schematic diagram of the C2f module.

Figure 6 illustrates the schematic diagram of the C2f module and the bottleneck of the proposed model. The C2f module is utilized to improve quality after feature fusion. It is known as an improvement of the original C3 module, which is considered a benefit of the YOLOv8 model with high gradient information. It decreases one convolutional layer and makes use of the bottleneck module to extend the gradient branch to acquire high gradient flow data when ensuring lightweight. The main idea of the given structure is to improve time of multi-scale fusion and the probability of attaining a higher detection accuracy rate. Since the model is lightweight, B3-N3 and B4-N4 are included, and one unit is used. The respective process can be explained below.


$$\begin{array}{c} {\text{Net}}_{5}^{\text{out}}=\text{C2f }(\text{Concate }(\text{Conv }({\text{Net}}_{5}^{\text{in}}),{\text{ B}}_{5}^{\text{out}}),\text{ n}) \end{array}$$

$$\begin{array}{l} {\text{Net}}_{\text{in}}^{\text{out}}=\text{C2f }\left(\text{Concate }\left(\text{Conv }\left({\text{Net}}_{\text{i}}^{\text{in}}\right),{\text{ B}}_{\text{in}}^{\text{out}},{\text{ P}}_{\text{in}}^{\text{out}}\right),\text{ n}\right) \end{array}$$

where Conv and C2f are represented as conforming module operations. Net, B, and P corresponded to backbone feature maps, as PAN and FPN, respectively; n denotes the number of C2f uses. On the other hand, the attention mechanism called Decoupled Fully Connected (DFC) avoids the prevailing attention algorithms for feature capture at long distances and computational complexity. Its execution is deliberated in Equations (1) and (2).


(1) $$\begin{array}{c} \text{Y}\prime \;=\text{ X}{\;}^{*}\text{Fea}{\text{t}}_{1^{*}1} \end{array}$$

(2) $$\begin{array}{c} \text{Y }=\text{ Concat}\left([\text{Y}\prime ,\text{ Y}\prime *\text{Fea}{\text{t}}_{\text{dep}}]\right) \end{array}$$

where Feat_depis_ represented as a depth-wise convolution. The depth-wise convolution acts upon feature maps to attain a linear transformation process. In addition, the DFC attention mechanism is used directly as a deeply separable structure, with the simplest structure being to acquire an attention map along with information. The particular measuring process is expressed in the following Equations (3) and (4):


(3) $$\begin{array}{l} \propto_{\text{hw}}^{\prime }\;=\;\overset{\text{H}}{\underset{{\text{h}}^{\prime }=1}{\sum }}{\text{Feat}}_{\text{h,h}\prime ,\text{w}}^{\text{H}}⊙{\text{X}}_{{\text{h}}^{\prime }{\text{w}}^{\prime }}\text{h }=\text{ 1},\text{ 2},\;...,\text{ H, w }=\text{ 1, 2},\;.\;.\;.\;,\text{W} \end{array}$$

(4) $$\begin{array}{c} \propto_{\text{hw}}\;=\;\overset{\text{W}}{\underset{\text{w}\prime =1}{\sum }}\text{Fea}{\text{t}}_{\text{w},\text{h},{\text{w}}^{\prime }}^{\text{W}}\;⊙\propto_{{\text{hw}}^{\prime }}^{\prime }\text{ h}=1,2,...,\text{H},\text{ w}=1,2,\ldots ,\text{W} \end{array}$$

where Feat refers to the depth-wise separable convolution process, which is classified into vertical and horizontal directions; $\propto_{\;}^{\prime }$ is an attention map in the vertical direction and ∝ is an attention map on $\propto_{\;}^{\prime }$ in the horizontal direction. Therefore, feature vectors are transformed linearly to estimate three matrices, namely V, K, and Q. The calculation process is expressed in Equations (5)–(7).


(5) $$\begin{array}{c} \text{Q}=\;{\text{X}}^{\text{rev}}\text{ Wi}{\text{d}}^{\text{Q}} \end{array}$$

(6) $$\begin{array}{c} \text{K }=\;{\text{X}}^{\text{rev}}\text{Wi}{\text{d}}^{\text{rev}} \end{array}$$

(7) $$\begin{array}{c} \text{V}=\text{X}\prime \text{Wi}{\text{d}}^{\text{V}} \end{array}$$

Then attention to relation from place to place is obtained through structuring a directed graph to localize its related region. The particular implementation is as follows: V and Q for each place are processed by region to obtain the levels Q^r^ and K^r^. After that, Q^r^ and K^r^ dot products are estimated to obtain the adjacency matrix and *Adj*^*rev*^ is used to calculate the inter-place correlation, and the equation is


$$\begin{array}{c} \text{Ad}{\text{j}}^{\text{rev}}\;=\;{\text{Q}}^{\text{rev}}\;{\left({\text{K}}^{\text{rev}}\;\right)}^{\text{T}} \end{array}$$

Further, *Adj*^*rev*^ is clipped and the minimum relevant token in *Adj*^*rev*^ is filtered out at a coarse-grained level. The top k relevant regions in *Adj*^*rev*^ have been recollected to acquire a matrix of routing index, *I*^*rev*^ which is deliberated in Equation (8).


(8) $$\begin{array}{c} {\text{I}}^{\text{rev}}\;=\text{ topkIndex}\left({\text{A}}^{\text{rev}}\;\right) \end{array}$$

Subsequently, at a fine-grained level, token-to-token attention is utilized. This attention is focused on k routing regions that are indexed and collecting all the V and K tensors in k regions to obtain *V*^*foc*^ and *K*^*foc*^ that are calculated through Equations (9) and (10).


(9) $$\begin{array}{c} {\text{K}}^{\text{foc}}=\text{ focal}\left(\text{K},\;{\text{I}}^{\text{rev}}\right) \end{array}$$

(10) $$\begin{array}{c} {\text{V}}^{\text{foc}}\;=\text{ focal}\left(\text{V},\;{\text{I}}^{\text{rev}}\;\right) \end{array}$$

The collected *V*^*foc*^ and *K*^*foc*^ are processed along with the attention. A Local Context Enhancement (LCE) term is employed to obtain the output tensor. The formula is deliberated in Equation (11).


(11) $$\begin{array}{c} \text{Out}=\text{ Attention}\left(\text{Q},\;{\text{K}}^{\text{foc}},\;{\text{V}}^{\text{foc}}\;\right)+\text{ LCE }\left(\text{V}\right) \end{array}$$

For the size of the input feature *h* × *w* × *c*, the required SFFs are utilizing the size of regular convolution *k* × *k* in Equation (12). C represents the number of channels of input data.


(12) $$\begin{array}{c} \text{SFF}{\text{s}}_{\text{Conv}}\;=\text{ h }\times \text{ w }\times \;{\text{k}}^{2}\;\times \;{\text{c}}^{2} \end{array}$$

A deep convolutional kernel makes the sliding operations over the input channel space to estimate the output channel features. The SFFs for deep convolution are estimated as the output channel features that are calculated through Equation (13).


(13) $$\begin{array}{c} \text{SFF}{\text{s}}_{\text{DWConv}}=\text{ h }\times \text{ w }\times \;{\text{k}}^{2}\;\times \text{ c} \end{array}$$

Correspondingly, deep convolution requires other computational costs and point-by-point convolution to compensate for decreasing accuracy after the convolution function. It introduces Partial Convolution (PConv), which utilizes regular convolution to process the operation on continuous features in the input channel. ${\text{c}}_{\text{p}}^{\;}$ is the channel number in input features. The formula for measuring SFFs of pConv is indicated in Equation (14).


(14) $$\begin{array}{c} \text{SFF}{\;}_{\text{sPConv}}=\text{ h }\times \text{ w }\times \;{\text{k}}^{2}\;\times \;{\text{c}}_{\text{p}}^{2} \end{array}$$

${\text{c}}_{\text{p}}^{\;}$, $\frac{1}{4}$ of a number of feature channels (input) c, the SFFs of pConv are 1/16 of the conventional convolution. This decreases the number of memory accesses when minimizing the parameters. Firstly, pConv has been utilized to swap the two 1 x 1 convolutional layers in the SFF block, which enhances the receptive field when making the original module more efficient and faster. Secondly, residual concatenation has been added to the last two convolutional layers in the block to develop the output information features and decrease the effective feature loss. It can optimize the present model's performance. Due to the anchor-free idea usage, the YOLOv8 loss function is greatly different from the previous YOLOv5 series. The optimization was comprised of two parts, namely regression and classification. The regression part utilizes the Boundary Box Regression Loss (BBRL) and Distribution Focal Loss (DFL), and the classification loss utilizes Binary Cross Entropy Loss (BCEL). The loss function is expressed in the following Equation (15):


(15) $$\begin{array}{c} \text{fun}{\text{c}}_{\text{loss}}\;=\;{\text{λ}}_{1}\text{fun}{\text{c}}_{\text{BCEL}}\;+\;{\text{λ}}_{2}\text{fun}{\text{c}}_{\text{DFCL}}\;+\;{\text{λ}}_{3}\text{ fun}{\text{c}}_{\text{BBRL}}\\ \;+\;{\text{λ}}_{3}\text{ fun}{\text{c}}_{\text{Out}} \end{array}$$

The loss of prediction set is necessarily the cross-entropy loss, and the calculation can be deliberated in Equation (16).


(16) $$\begin{array}{c} \text{fun}{\text{c}}_{\text{BCEL}}\;=\text{ weg}\left[\text{class}\right]\left(-\text{x}\left[\text{cls}\right]+\text{log}\left(\underset{\text{j}}{\sum }\exp\left(\text{x}\left[\text{j}\right]\right)\right)\right) \end{array}$$

where cls is a number of categories, *weg*[*class*] refers to each class weight, and x refers to the probability value after the sigmoid activation. DFL is an optimization of the focal loss function that generalizes discrete outcomes of classification into unremitting outcomes through integration. Hence, the calculation is expressed in the following Equation (17).


(17) $$\begin{array}{c} \text{fun}{\text{c}}_{\text{DFL}}\left({\text{S}}_{\text{i}},\;{\text{S}}_{\text{i}+1}\right)\\ =\;-\left(\left({\text{y}}_{\text{i}+1}-\text{ y}\right)\log\left(\text{Si}\right)+\left(\text{y }-\;{\text{y}}_{\text{i}}\right)\log\left({\text{S}}_{\text{i}+1}\right)\right) \end{array}$$

where *y*_*i*_ and *y*_*i*+1_ represent values from the left and right sides that are near the consecutive labels y. Simultaneously, while the prediction box contains a high degree of coincidence with the target box, the loss function made the model obtain better generalization capability with less training intervention through weakening the geometric factors penalty. A 2-layer attention mechanism and active non-monotonic FM apparatus are utilized. The expression is provided in Equations (18) and (19).


(18) $$\begin{array}{c} \text{fun}{\text{c}}_{\text{BBRL}}\\ =\;\left(\text{1}-\frac{{\text{Wid}}_{\text{i}}{\text{Heg}}_{\text{i}}}{\text{Su}}\right)\text{ exp}\left(\;\frac{{\left({\text{x}}_{\text{p}}\;-\;{\text{x}}_{\text{gt}}\right)}^{2}\;+{\left({\text{y}}_{\text{p}}\;-\;{\text{y}}_{\text{gt}}\right)}^{2}}{{\left(\text{Wi}{\text{d}}_{\text{g}}^{2}\;+\text{ He}{\text{g}}_{\text{g}}^{2}\;\right)}^{{\;}^{*}}}\;\right)\text{γ} \end{array}$$

(19) $$\begin{array}{c} \text{γ}=\frac{\text{β}}{{\text{δα}}^{\text{β }-\text{ δ}}} \end{array}$$

where β refers to the abnormality degree of the predicted box. The small degree of abnormality denotes that the anchor box quality is higher. Henceforth, β is used to build the non-monotonic focal number assigned small gradient gains for predicting large outliers' boxes. α and δ are referred to as hyperparameters; *x*_*p*_ and *y*_*p*_ denote coordinate values of the prediction box, *x*_*gt*_ and *Y*_*gt*_ represent ground truth coordinate values. The corresponding *wid* and *heg* values denote the width and height of the two boxes, respectively. The process can be expressed as


$$\begin{array}{c} {\text{S}}_{\text{u}}\;=\text{ widheg }+\text{ wi}{\text{d}}_{\text{gt}}\text{he}{\text{g}}_{\text{gt}}-\text{Wi}{\text{d}}_{\text{i}}\text{He}{\text{g}}_{\text{i}}. \end{array}$$

#### 3.5.3 SFF

The Selection Focal Fusion (SFF) block is utilized in the proposed research. It is a technique utilized in the YOLOv8 model that is popular in object detection algorithms. It improves the accuracy of detection by selectively fusing features from three layers of the network. In the YOLOv8 model, a network architecture consists of multiple detection layers at various scales. Each of the detection layers is accountable for detecting objects of various sizes. SFF is applied to three detection layers and employs an attention mechanism for featuring the image data to improve their performance. Furthermore, focal aggregation classifies the images at both horizontal and vertical levels and provides the attention mechanism that is presented for the optimization of the backbone network that enhances the attention of the model to critical information. The proposed SFF substantially decreases the missed detection rates of small objects and improves the detection performance of the present model.

#### 3.5.4 Prediction phase

The prediction phase is a commonly used technique by researchers to determine the efficiency of the proposed system. In the prediction phase, the algorithm is processed using the test data, which will reveal the performance of the present model. Finally, the system's efficiency is calculated using certain performance matrices, such as mAP, F1-score, precision, and recall, to evaluate the efficiency of the projected model.

## 4 Results

### 4.1 EDA

**Figure 7:**
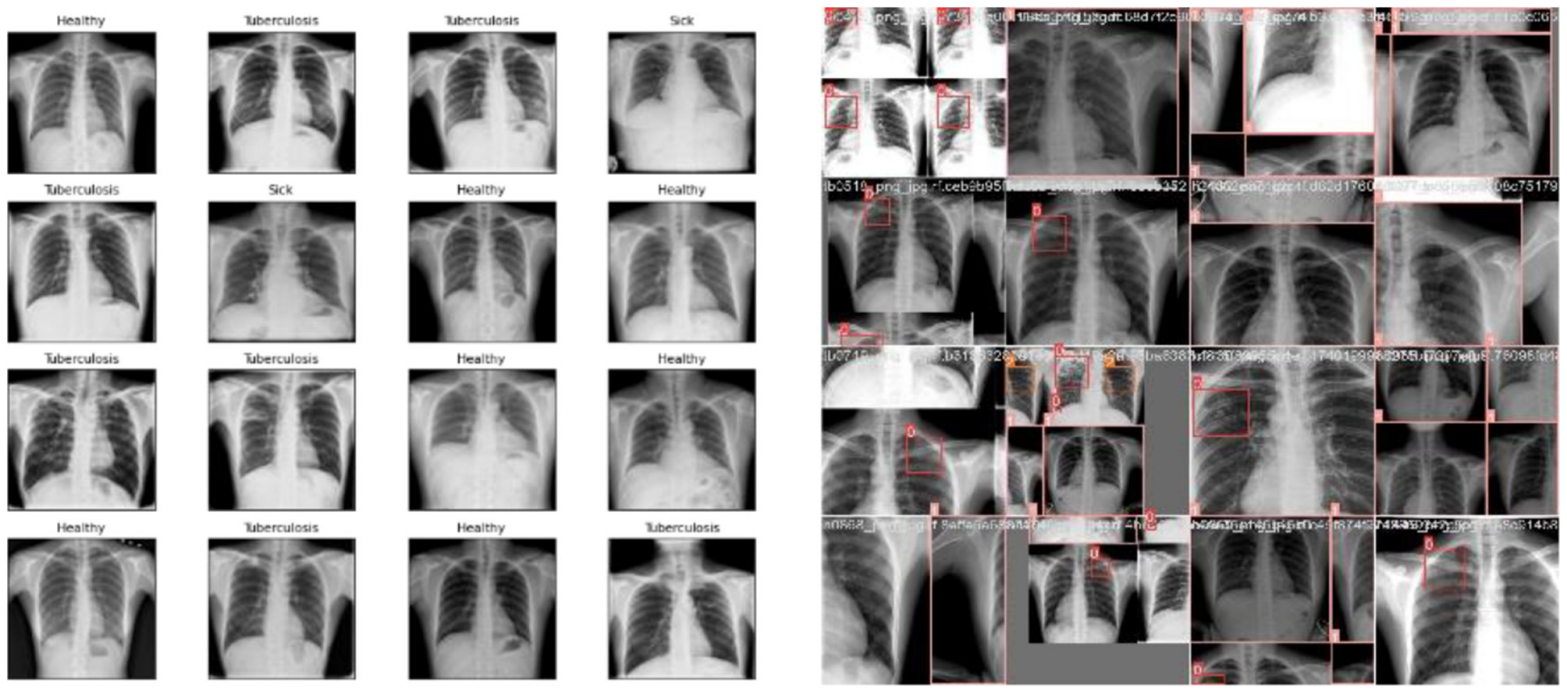
Train batch images.

**Exploratory Data Analysis** (EDA) supports examining the process of data comprehension in detail and aids in learning different characteristics of data. It helps in representing the data statistically. Various visual representations are deliberated for the identification of patterns and styles, such as heat maps, histograms, box plots, bar charts, and scatter plots. Furthermore, EDA is significantly utilized for outliers, anomalies, error detection, and other suspicious patterns or assumptions in the data. Therefore, it can be utilized for data comprehension before creating assumptions. Figure 7 shows the sample data used in the proposed research, which consists of lungs.

**Figure 8:**
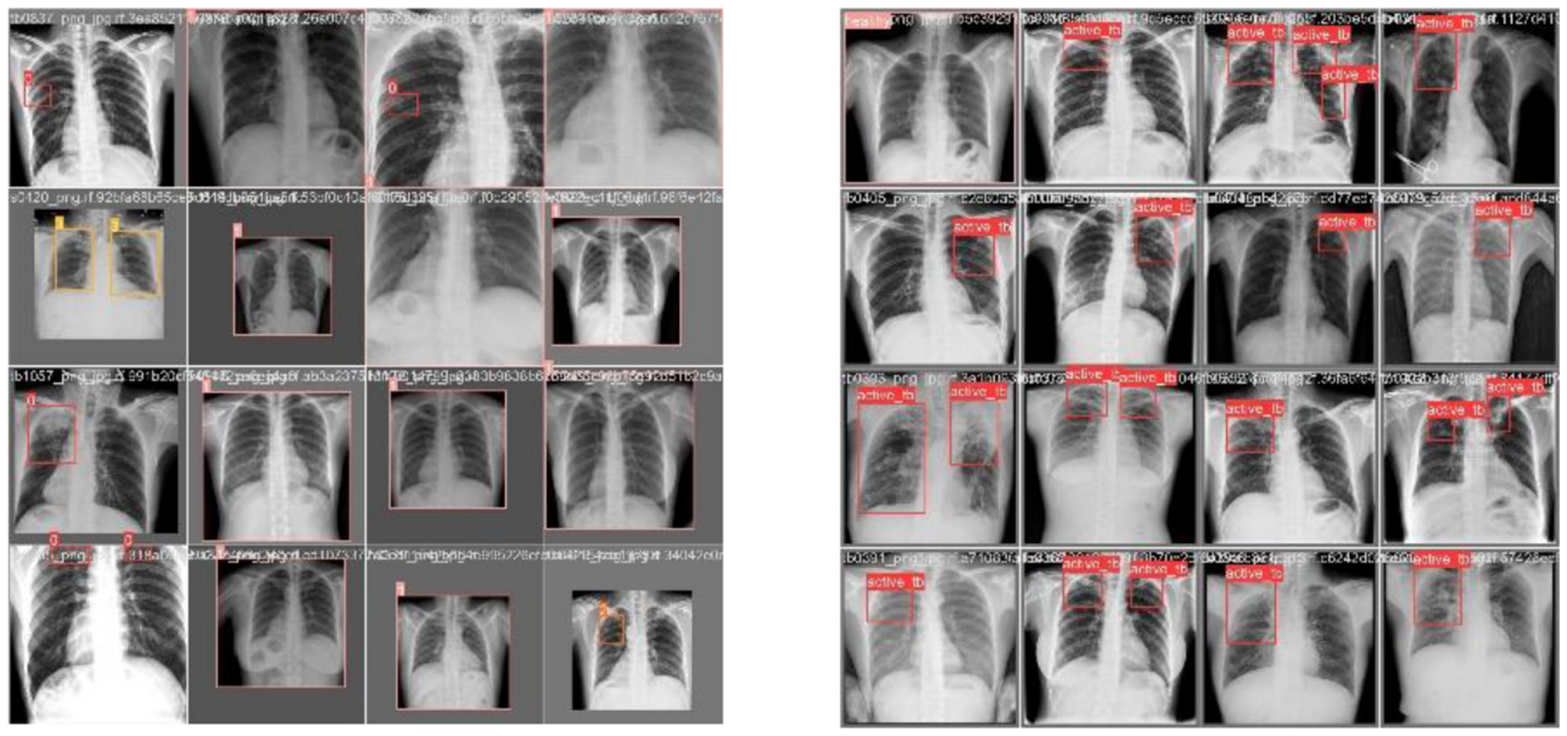
Validation batch images.

**Figure 9:**
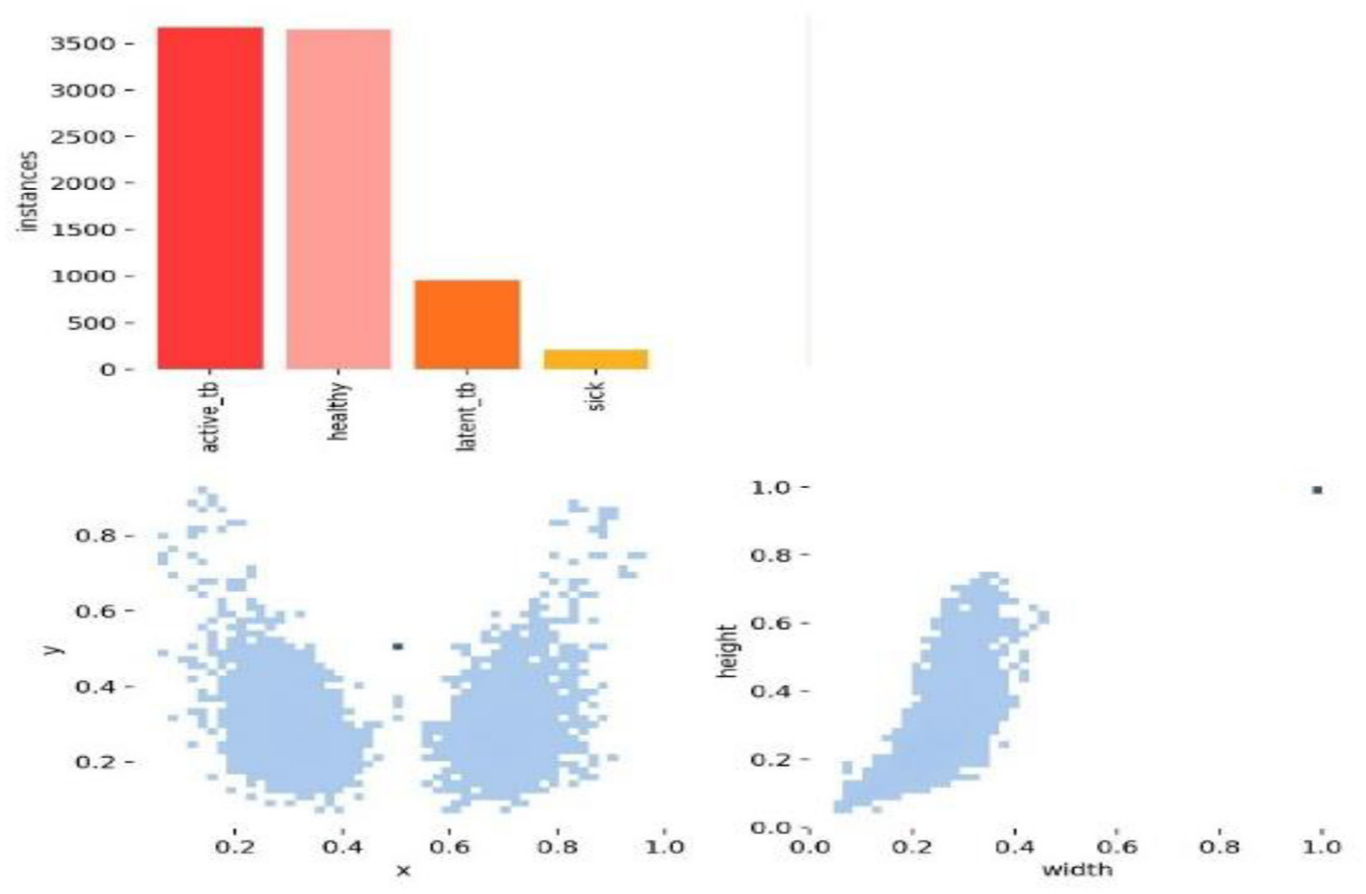
Labeling of objects in a dataset.

Figures 7, 8 deliberate the sets of training and validation of image data from the dataset. From Figure 8, the validation set of images shows the level of TB in the lungs; Figure 9 signifies the information about manually labeling objects in the dataset. Here, the red box indicates active TB, salmon pink indicates healthy, the yellow box indicates sick, and the orange box indicates latent TB.

**Figure 10:**
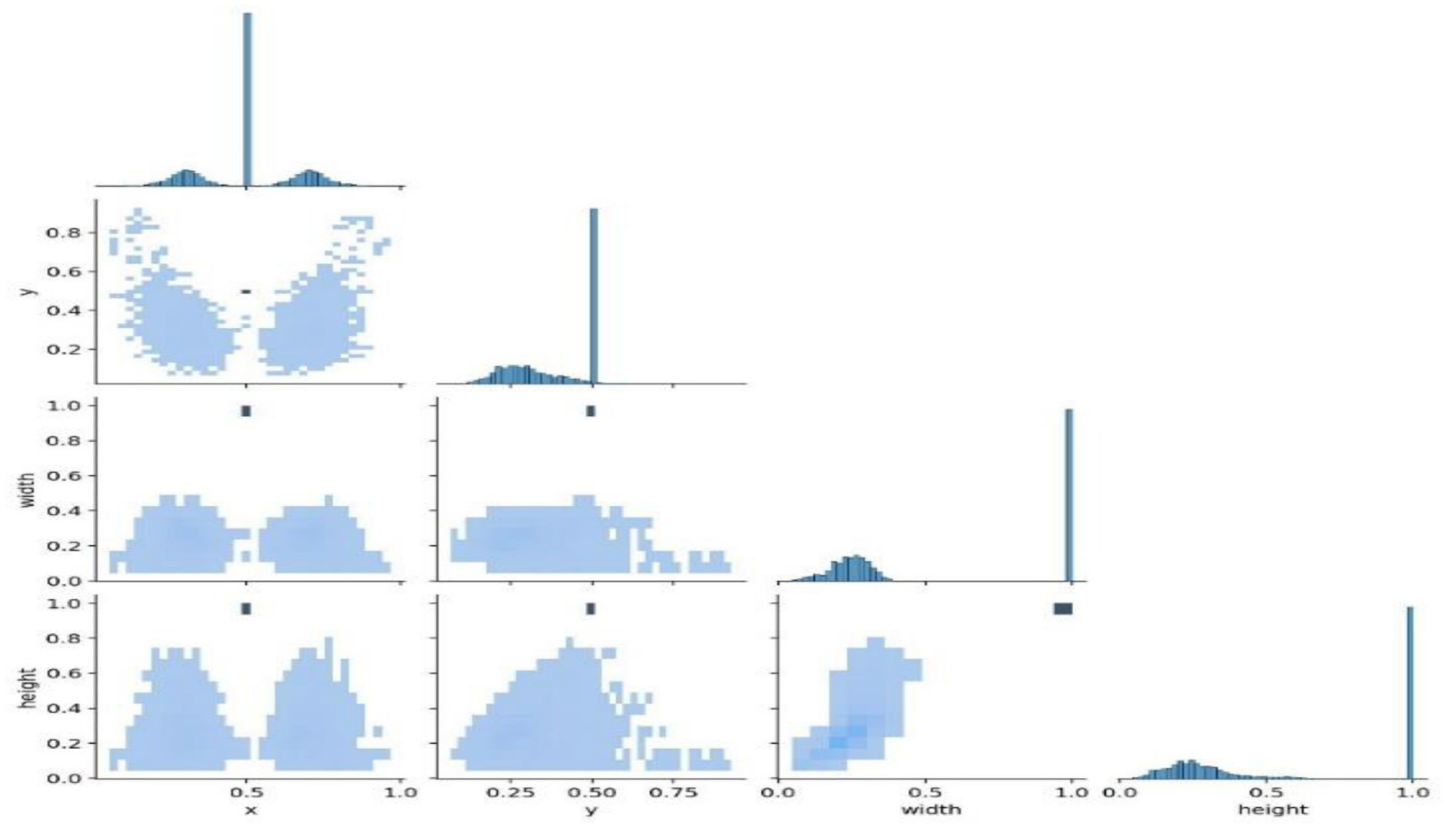
Correlogram label.

Figure 9 shows the analysis of the dataset, which is surmised. The dataset is comprised of a huge number of small objects that can exist in an uneven and dense distribution. The first subfigure illustrates the number of objects of each type in the dataset. The second subfigure shows the distribution of center point coordinates of objects in bounding boxes in the dataset. The third subfigure represents a scatter plot of the corresponding height and width of the object in the bounding box. Moreover, Figure 10 presents the correlogram label.

**Figure 11:**
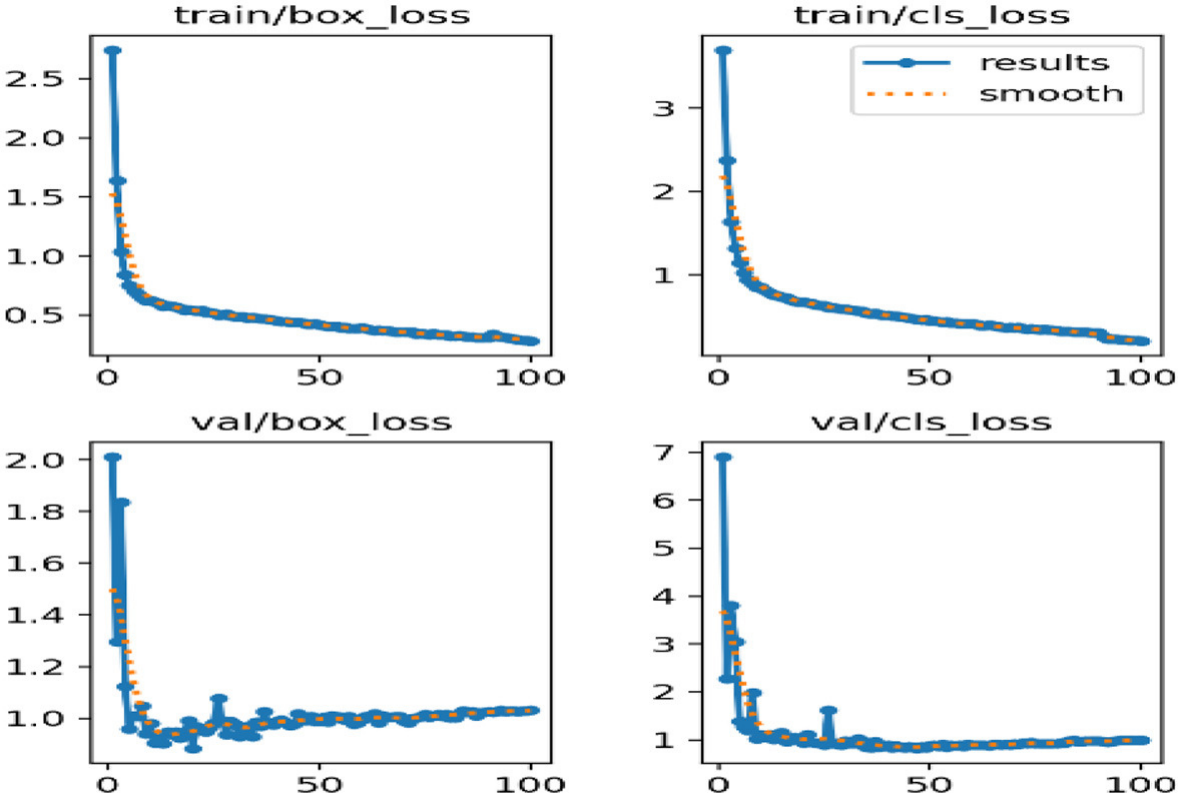
Curves of box classification and prediction loss for validating and training sets.

Figure 10 deliberates the correlogram label. A correlogram is known as a graphical representation that shows correlation coefficients among variables in the dataset. Moreover, the label in the correlogram denotes features or variables that are being examined. The YOLOv8 model endured training on the TBX-11k dataset for its challenging X-ray samples and limited data. Figure 9 shows the xywh of the dataset, where x and y are positions and h and w refer to height and width. The estimation has encompassed training and validation sets, which reveal the classification and box prediction loss on the validation set. Figure 11 represents the learning curves of box classification and prediction loss for training and validating the dataset.

Figure 11 depicts the learning curves of box classification and prediction loss for training and validating the dataset. To address the overfitting, the annotated data aimed to augment the size of the samples for improved generalization. During the training, it is important to notice that optimal weights are saved and the checkpoint weights are retained for deployment. Despite this, Figure 11 shows the better performance of the proposed model, attaining a prediction loss below 0.06 and a classification loss below 0.1 on the validation set, displaying its proficiency in localizing and identifying the lesions in lung images.

### 4.2 Performance metrics

Performance metrics are primarily used for observing the efficiency of the projected research by utilizing various metrics like recall rate, precision, mAP, and F1-score value.

#### 4.2.1 Recall

The term recall is deliberated as the reclusive of the production metric that estimates the total of accurate positive categories made out of all the optimistic categories. It is calculated with the following equation:


$$\begin{array}{c} \text{Recall}=\frac{\text{True\_Pos}}{\text{False\_Neg}+\text{True\_Pos}} \end{array}$$

#### 4.2.2 Precision

The term precision is denoted as the covariance unit of technique, which results from the appropriately recognized cases (*True*_*P*_*os*) to the total group of cases that are accurately categorized (*True*_*P*_*os* + *False*_*P*_*os*). It includes the repeatability and reproducibility of the capitals. Equation (20) depicts the formula for precision.


(20) $$\begin{array}{c} \text{Precision}=\frac{\text{True\_Pos}}{\text{False\_Neg}+\text{True\_Pos}} \end{array}$$

#### 4.2.3 F1-score

The F1-score is represented as a measure of the appropriate mean rate of recall and precision value. The mathematical equation for the F1-score is depicted in Equation (21).


(21) $$\begin{array}{c} \text{F}1\;-\text{ score }=\;2\;\times \frac{\text{Recall }\times \text{ Precision}}{\text{Recall }+\text{ Precision}} \end{array}$$

#### 4.2.4 mAP

Mean Average Precision (mAP) is computed utilizing Equation (2). It is a broadly accepted performance metric for models of object detection. The mAP is evaluated by considering the mean of AP for each of the classes. AP for each class k is determined by measuring the area under the precision-recall curve. mAP offers a single score that considers precision, recall, and IoU, avoiding bias in performance.


$$\begin{array}{c} \text{mAP}=\frac{1}{\text{n}}\;\overset{\text{k}=\text{n}}{\underset{\text{k}=1}{\sum }}\text{A}{\text{P}}_{\text{k}}\begin{array}{c} \;\\ \; \end{array} \end{array}$$

### 4.3 Experimental results

**Figure 12:**
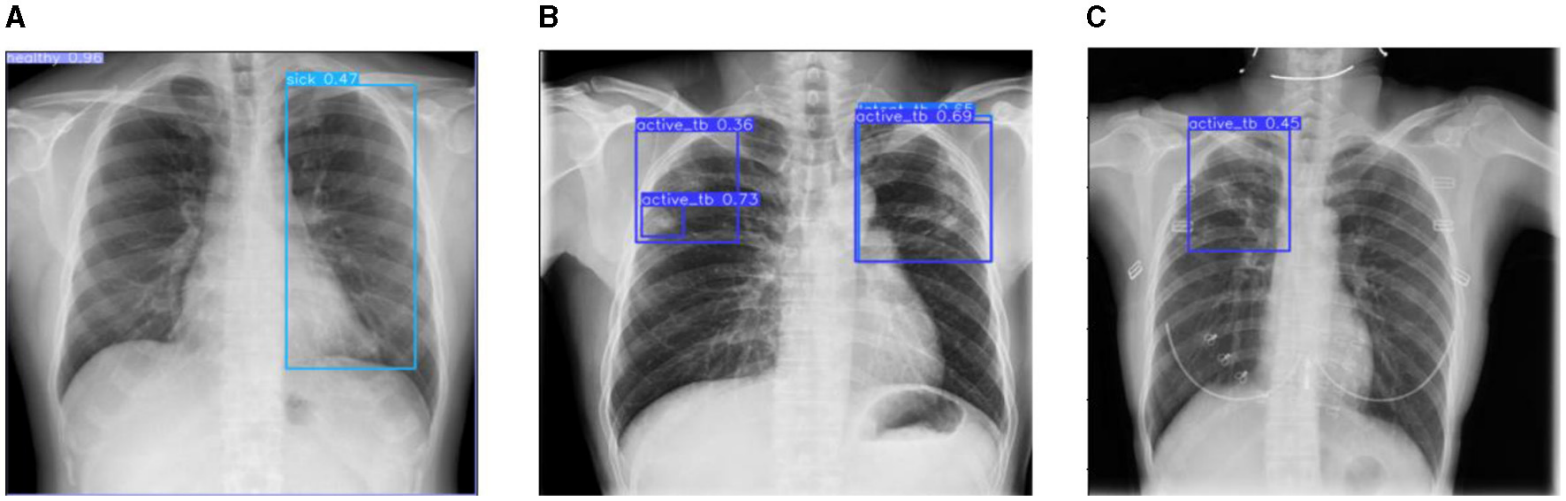
Prediction results of existing YOLOv8 model. **(A)** X-ray with both healthy and sick regions. **(B, C)** X-ray with active TB and latent TB.

**Figure 13:**
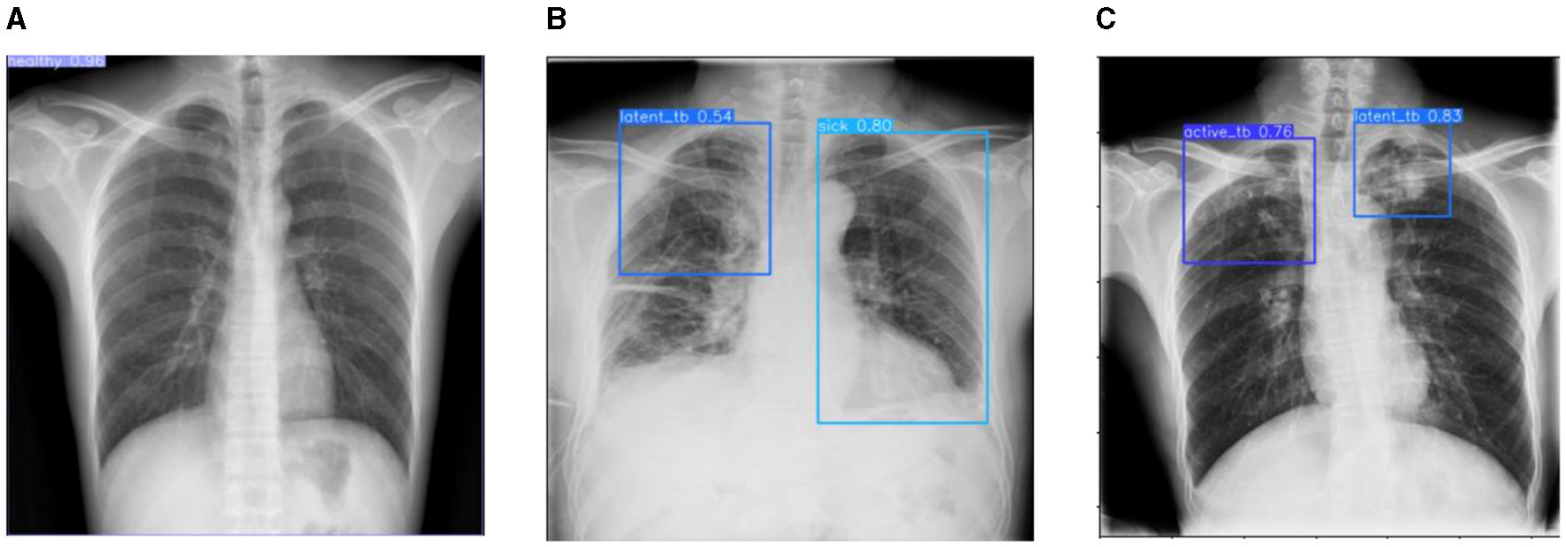
Prediction results of the proposed YOLOv8 model. **(A)** X-ray with healthy lung. **(B)** X-ray with sick region. **(C)** X-ray with latent TB.

This section discusses the results accomplished by the proposed mechanism for predicting Active TB, Sick but non-TB, Healthy, and Latent TB with the Kaggle TBX-11k dataset. Furthermore, the outcomes obtained in the internal comparison of the present research YOLOv8 model and existing data. Figures 12, 13 signify various illustrations of lungs and the prediction results of the proposed research.

Figure 12 represents the existing sample image data from the TBX-11k dataset. After the implementation, the prediction results are shown in Figure 13. It predicted active TB, latent TB, sick, and healthy among the X-ray images. Here, the lavender box indicates healthy, the light blue box indicates sick, the violet box indicates active TB, and the dark blue box indicates Latent TB in Figure 12. The results for both healthy and sick were 0.47%, which is less than the proposed model. However, Figure 13A shows a healthy lung, and (b) shows a sick lung with a 0.80% better prediction. The latent_TB in Figure 12 predicts 0.65% whereas, whereas latent_TB in Figure 13C results in 0.83% of. Likewise, the active_TB results in 0.36%, 0.73%, 0.69%, and 0.45% in Figure 12, whereas the proposed model in Figure 13 results in 0.76%, which shows a higher prediction than the existing model. While validating, the results are attained at 0.2 ms of speed, 2.0 ms of inference of pre-process, 0.0 ms of loss, and 1.1 ms of post-process per image. Furthermore, Table 2 depicts the validating results of the proposed system.

**Table 2:** Validating results of proposed system.

**Class**	**Images**	**Instances**	**Box (P)**	**R**	**mAP50**	**mAP50-95**
All	357	449	0.711	0.622	0.657	0.475
Active_TB	357	207	0.773	0.56	0.676	0.32
Healthy	357	182	1	0.995	0.995	0.994
Latent_TB	357	41	0.176	0.146	0.107	0.0567
Sick	357	19	0.895	0.789	0.849	0.529

Table 2 illustrates the proposed system for validating results. It highlights the overall prediction results that comprised 449 instances, 0.711 of precision, 0.622 of recall, 0.657 of mAP50, and 0.475 of mAP50-95. Additionally, it shows results for predictions such as active TB, Latent TB, healthy, and sick.

### 4.4 Comparative analysis

The section illustrates the comparative analysis of the proposed mechanism with the existing approaches depending on the performance metrics. Table 3 deliberates the comparative analysis of YOLOv8 with the YOLOv7 model at mAP values.

**Table 3:** Comparative analysis of proposed research (Bista et al., 2023).

**Model**		**All classes (mAP@0.5)**
YOLOv7	Base model with class imbalance	0.249
	Base model with image weights	0.211
	Base model along with minority class image augmentation	0.280
	Putting it all together and evolving the hyperparameter	0.587
YOLOv8	Base model	0.627
	Selective Focal Fusion (SFF) Block in YOLOv8	0.657

From Table 3, the base model of YOLOv8 has attained 0.627 mAP values, whereas the proposed model of SFF in YOLOv8 has attained 0.657 mAP values. It is proven that the present model attains 0.37 higher than the base model, which represents its efficiency. Additionally, Table 4 discusses the comparative outcomes of the proposed system.

**Table 4:** Comparative outcomes of proposed system.

**All classes at mAP**	**Proposed model**	**Existing model**
F1 confidence	0.66	0.63
Precision confidence	1	0.96
Recall confidence	0.76	0.71
Precision-recall confidence	0.65	0.62

**Figure 14:**
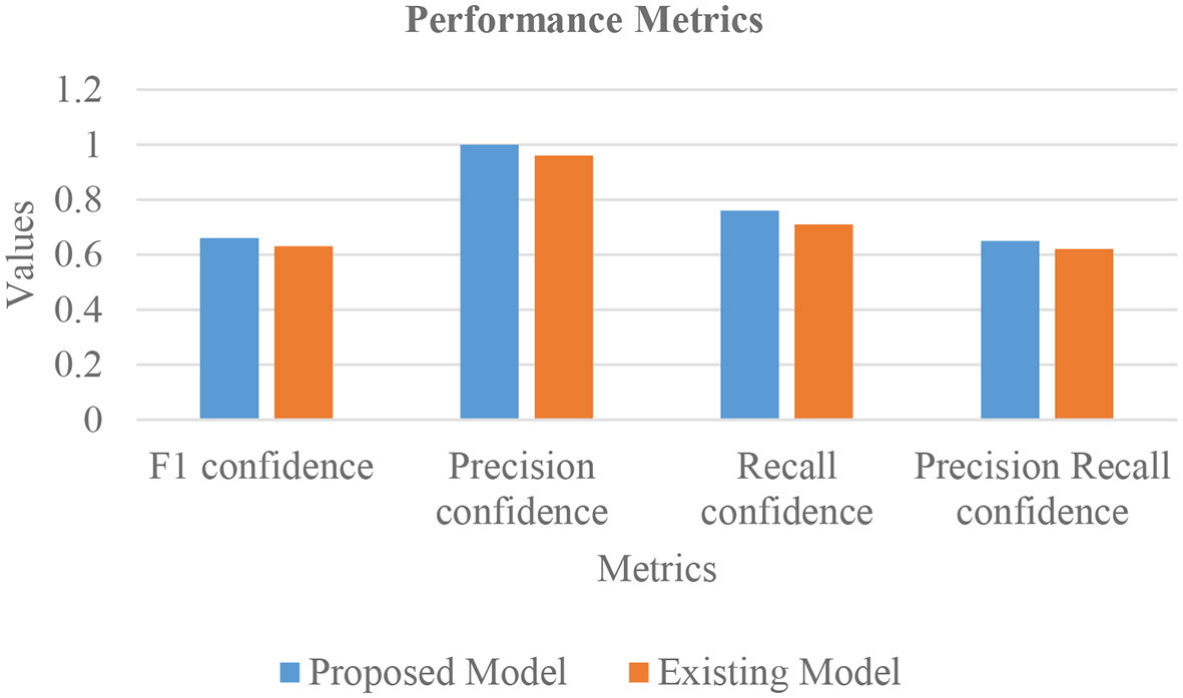
Performance metrics comparison.

In Table 4, the comparative outcomes of the existing and proposed models are presented. It shows the proposed research has attained better outcomes than the prevailing model in all classes at mAP values. It attained 0.03 of F1 confidence, 0.04 of precision confidence, 0.05 of recall confidence, and 0.03 of precision-recall confidence more than the prevailing model. Additionally, Figure 14 deliberates the performance metrics comparison.

Figure 14 illustrates that the proposed model attained better values than the prevailing model in all classes at mAP values. It attained 0.66 of F1 confidence, 1 of precision confidence, 0.76 of Recall confidence, and 0.65 of precision-recall confidence, which is more than the prevailing model values.

### 4.5 Performance analysis

The performance of the proposed algorithm is examined using evaluation metrics like Recall, Precision, F1-score, and accuracy. Likewise, a Confusion Matrix (CM) is utilized for identifying the performance of the proposed research. It encapsulates and envisages the performance of the classification algorithm. Hence, the CM signifies how many predictions are right and wrong as per the class. Figure 8 deliberates the CM of the respective system.

**Figure 15:**
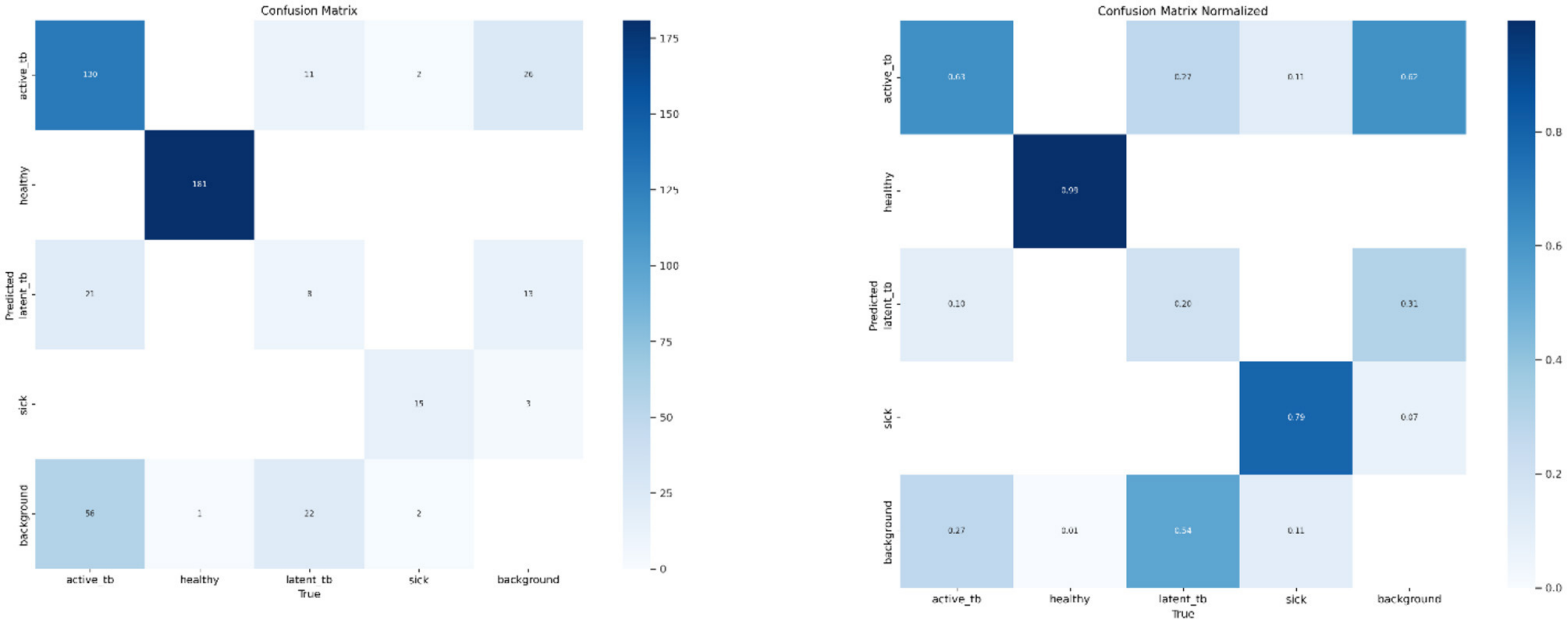
Confusion matrix and normalized confusion matrix.

**Figure 16:**
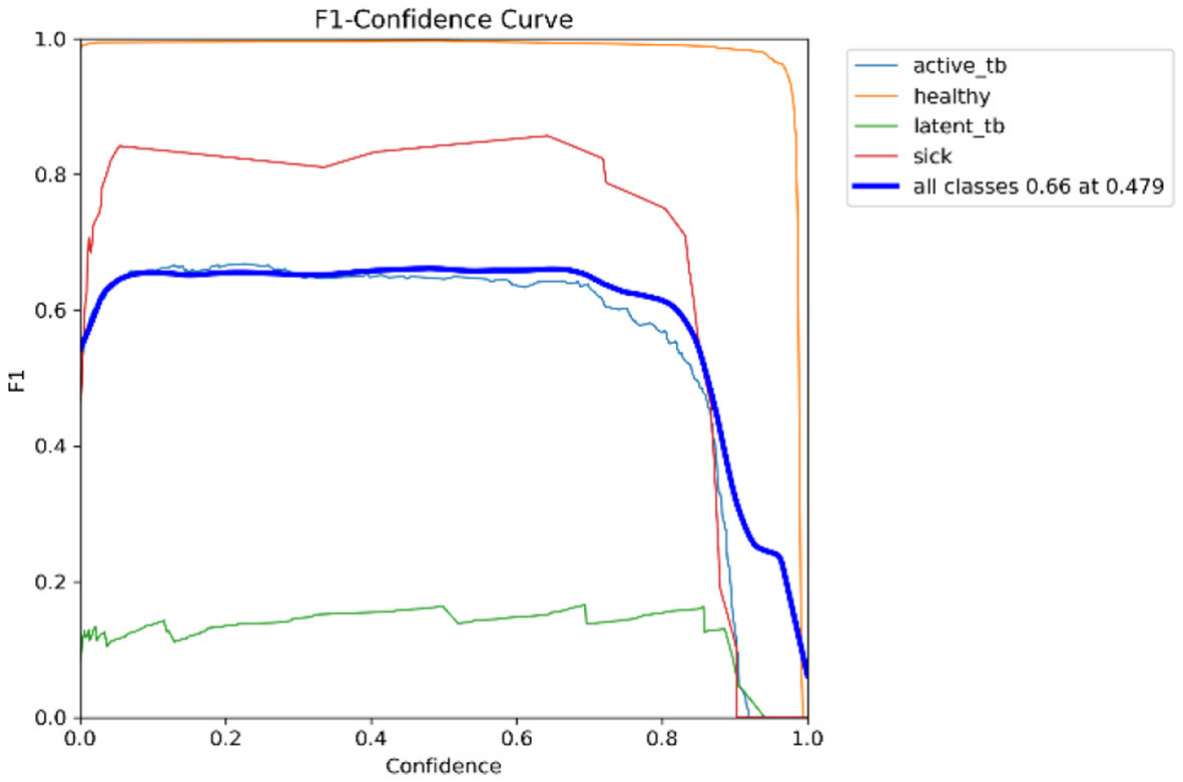
F1-curve.

**Figure 17:**
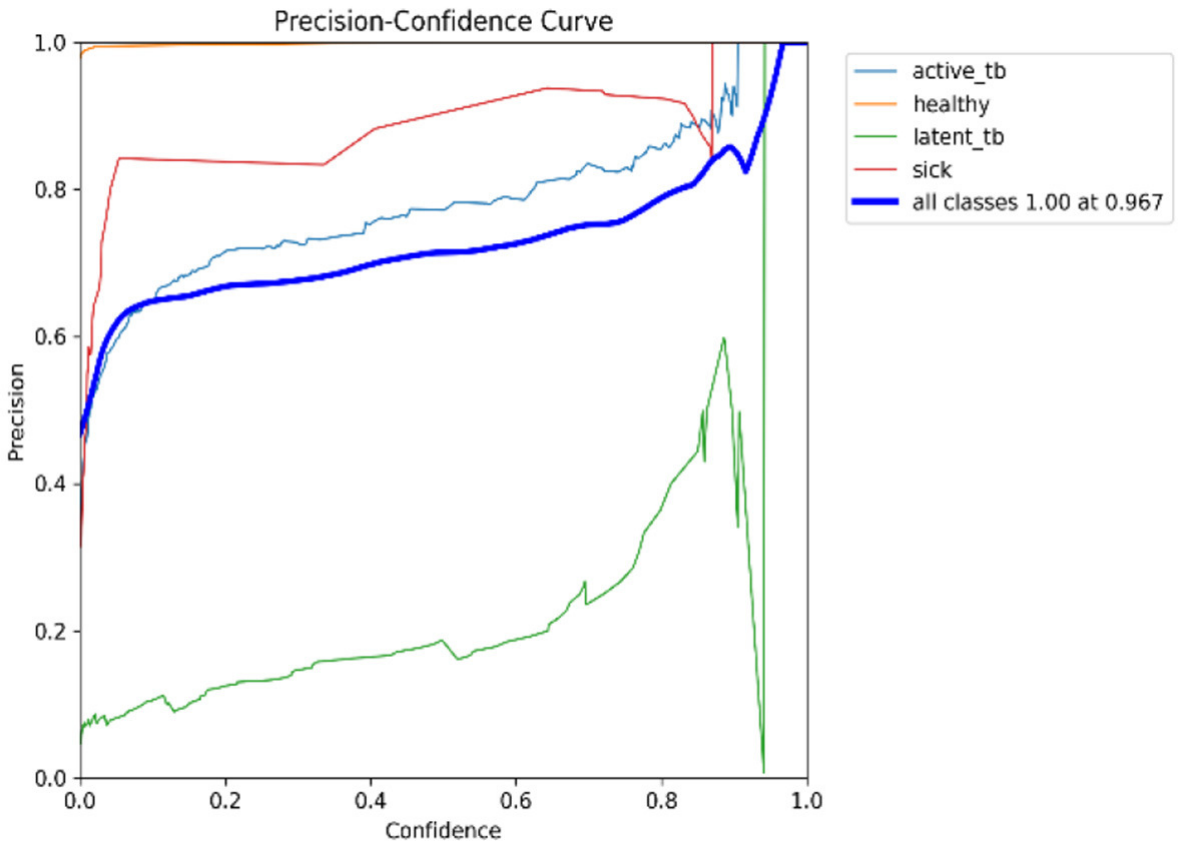
P-curve.

Figure 15 shows the CM of the proposed research. It represents the actual and correct prediction of the model. Figures 16, 17 show the F1 confidence curve and Precision confidence curve, respectively. Correspondingly, the confidence curves are associated with performance metrics that illustrate the performance of the proposed mechanism at various confidence levels.

**Figure 18:**
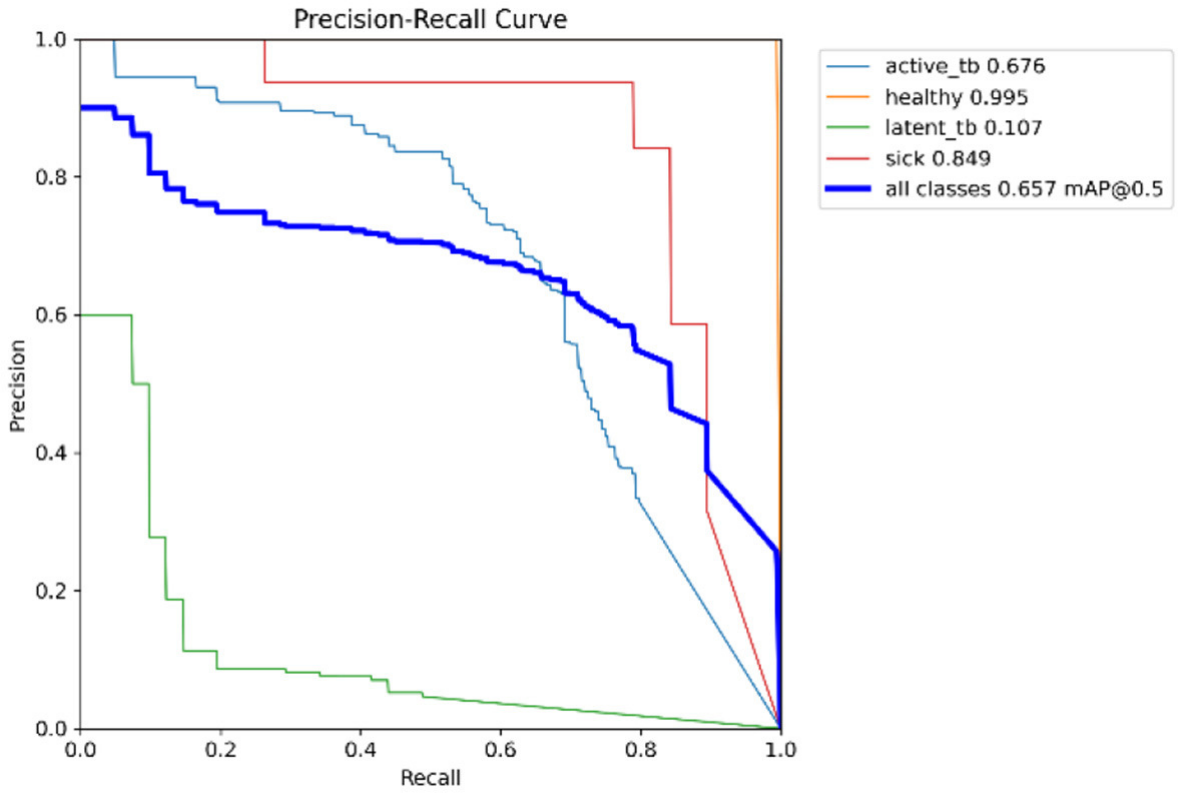
PR-curve.

**Figure 19:**
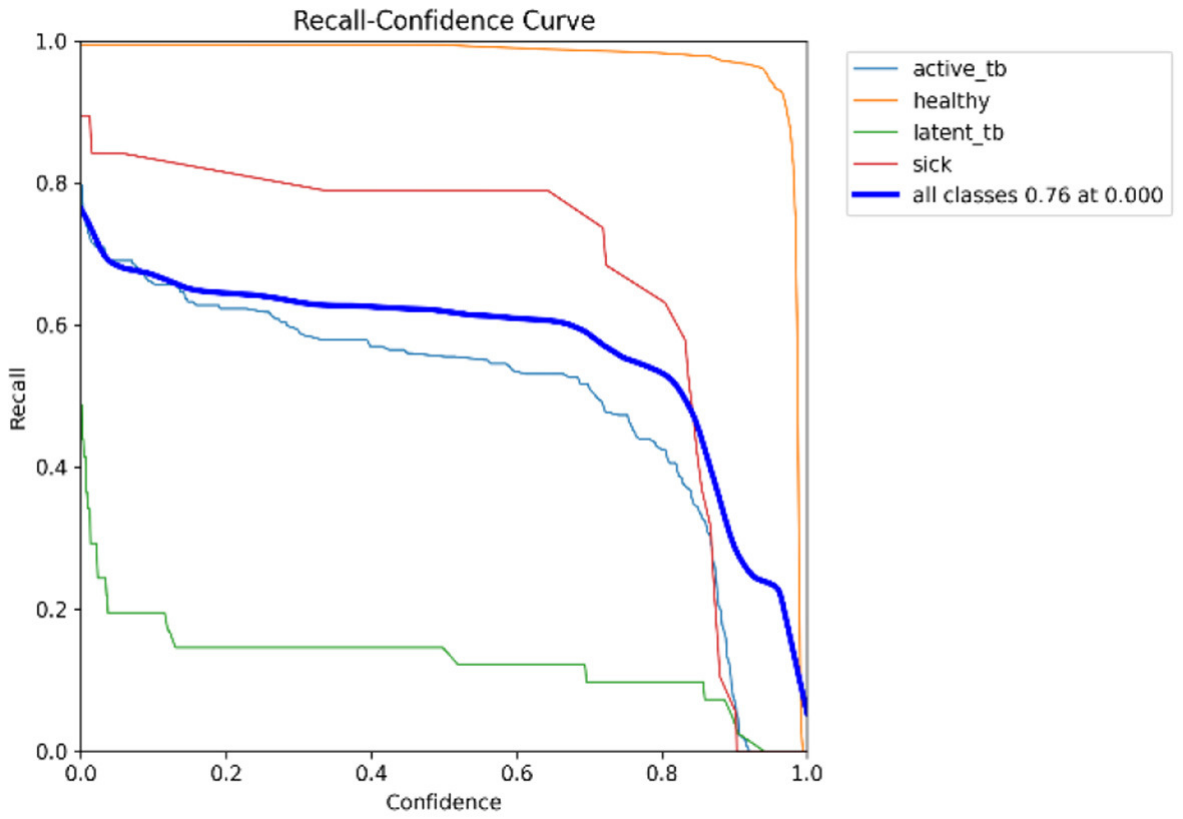
R-curve.

The F1-score is a measurement of the accuracy of the proposed model in classification tasks, taking into account both recall and precision. It combines these two metrics into a single value to obtain a balanced evaluation. Figure 17 deliberates the precision confidence curve; which focuses on positive prediction proportions made by the proposed research. The *p*-confidence curve shows the relationship between the precision value and the threshold value. Similarly, Figure 18 refers to the precision-recall curve, and Figure 19 exemplifies the recall-confidence curve.

Figure 18 deliberates the trade-off between recall and precision. Precision refers to the proportions of correctly classified positive instances out of all instances that are classified as positive. Recall refers to the correctly classified positive instances proportions out of the entire actual positive instances. The PR curve shows how the precision and recall points change as the classification threshold value varies. Similarly, in Figure 19, the recall-confidence curve denotes the association between the confidence threshold and the recall of the proposed research. They both help in assessing the ability of the proposed research to identify the positive instances correctly and provide a precise understanding of its confidence threshold performance.

## 5 Discussion

The contributions of the respective research and the utilized dataset are indicated in this section. Various types of research are focused on the prediction of TB through its CXR images (Ekins and Freundlich, 2011; Acharya et al., 2022), cough, and different tests like blood tests, sputum tests, imaging studies, skin tests (Maipan-uku et al., 2024), etc. In order to decrease the severity of the disease, it is important to diagnose the disease at an early stage by predicting TB. According to that, the primary cause of TB is Mycobacteria, which affects the human respiratory system. All the functions of the lungs will be affected, like chronic coughs, sneezes, sweating, chest pain, and fever. Correspondingly, predicting tuberculosis is necessary to consider a precise treatment to reduce the disease's consequences. For this purpose, the proposed mechanism focused on predicting Active TB, Latent TB, Healthy and Sick but non-TB through the Kaggle TBX-11k dataset. This dataset is comprised of chest radiograph samples that are related to TB and non-TB. Moreover, mAP is the prime metric for object detection. The proposed prediction model, with the advantage of efficient results from a smaller dataset, attains an mAP of 0.657, which is higher than other prevailing models. Besides, the dataset used is insufficient for prevailing research. For this reason, the respective model is examined with the traditional models, which reveal the efficiency of the proposed research. In the comparison, the proposed method achieved higher mAP values than conventional models and can assist radiologists and experts in providing effective TB prediction. Besides, it is envisioned to improve the life quality of TB patients.

## 6 Conclusion

Tuberculosis is a pathogenic and deadly disease caused by bacterial Mycobacteria. It mostly affects the human respiratory system and can even lead to death. Many people have been affected by TB owing to inaccuracy, late diagnosis, and deficiency of treatment. Hence, early and accurate diagnosis is a significant solution to preventing and checking tuberculosis. Applying technologies to support the medical business plays a primary role in improving accuracy and speed in prediction methods. A CXR is a major diagnostic tool utilized to screen for disease. However, the visual inspection accuracy through human experts is time-consuming and limited. To resolve this problem, the proposed research on SFF in the YOLOv8 model aimed to overcome the limitations and improve the prediction accuracy for TB detection, thereby reducing transmission risks and facilitating early intervention. Correspondingly, the utilization of the YOLOv8 architecture enabled TB pattern prediction and accurate detection in CXR images. The TBX-11k dataset is used to demonstrate its effectiveness in distinguishing sick but non-TB, active TB, Latent TB, and healthy cases. The data imbalance is mitigated through data augmentation and class weights methods that are comprised of image augmentation and focal loss, which result in robust generalization and improved performance of the proposed framework. After integrating the SFF into the YOLOv8 model, the respective research findings indicate that the projected system attained a promising mAP value of 0.657 on the validation set, making it an assistive tool for radiologists. Constantly, the outcome of the comparative analysis signifies that the respective model has outperformed the existing models. Though the proposed model performed efficiently, it did not encompass the large data due to the limited resources available at the time, and limited data are considered limitations. The usage of a wide range of data and associating it to the analysis and understanding the TB characteristics and patterns can be reached in future research.

## Data availability statement

The original contributions presented in the study are included in the article/supplementary material, further inquiries can be directed to the corresponding author.

## Author contributions

MP: Conceptualization, Methodology, Supervision, Validation, Writing – original draft, Writing – review & editing. WS: Conceptualization, Formal analysis, Investigation, Methodology, Supervision, Validation, Writing – original draft, Writing – review & editing. AB: Conceptualization, Formal analysis, Methodology, Supervision, Validation, Writing – original draft, Writing – review & editing. MM: Conceptualization, Formal analysis, Investigation, Methodology, Supervision, Validation, Writing – original draft, Writing – review & editing.
